# Aging gene signature of memory CD8^+^ T cells is associated with neurocognitive functioning in Alzheimer’s disease

**DOI:** 10.1186/s12979-023-00396-y

**Published:** 2023-12-02

**Authors:** Juan Joseph Young, Hong-Jai Park, Minhyung Kim, Jennefer Par-Young, Hugh Bartlett, Hye Sun Kim, Serhan Unlu, Lais Osmani, Min Sun Shin, Richard Bucala, Christopher H. van Dyck, Heather Allore, Adam P. Mecca, Sungyong You, Insoo Kang

**Affiliations:** 1 Department of Psychiatry, Yale School of Medicine, 300 Cedar Street, New Haven, CT 06520 USA; 2 Cedars-Sinai Medical Center, Los Angeles, CA USA; 3 Rutgers Robert Wood Johnson Medical School, New Brunswick, NJ USA; 4 Yale School of Public Health, New Haven, CT USA; 5 Cleveland Clinic Fairview Hospital, Cleveland, OH USA

**Keywords:** Aging, Alzheimer’s disease, Senescence, T cell, Adaptive immunity, Transcriptomics

## Abstract

**Background:**

Memory CD8^+^ T cells expand with age. We previously demonstrated an age-associated expansion of effector memory (EM) CD8^+^ T cells expressing low levels of IL-7 receptor alpha (IL-7Rα^low^) and the presence of its gene signature (i.e., IL-7Rα^low^ aging genes) in peripheral blood of older adults without Alzheimer’s disease (AD). Considering age as the strongest risk factor for AD and the recent finding of EM CD8^+^ T cell expansion, mostly IL-7Rα^low^ cells, in AD, we investigated whether subjects with AD have alterations in IL-7Rα^low^ aging gene signature, especially in relation to genes possibly associated with AD and disease severity.

**Results:**

We identified a set of 29 candidate genes (i.e., putative AD genes) which could be differentially expressed in peripheral blood of patients with AD through the systematic search of publicly available datasets. Of the 29 putative AD genes, 9 genes (31%) were IL-7Rα^low^ aging genes (*P* < 0.001), suggesting the possible implication of IL-7Rα^low^ aging genes in AD. These findings were validated by RT-qPCR analysis of 40 genes, including 29 putative AD genes, additional 9 top IL-7R⍺^low^ aging but not the putative AD genes, and 2 inflammatory control genes in peripheral blood of cognitively normal persons (CN, 38 subjects) and patients with AD (40 mild cognitive impairment and 43 dementia subjects). The RT-qPCR results showed 8 differentially expressed genes between AD and CN groups; five (62.5%) of which were top IL-7Rα^low^ aging genes (*FGFBP2*, *GZMH*, *NUAK1*, *PRSS23*, *TGFBR3*) not previously reported to be altered in AD. Unbiased clustering analysis revealed 3 clusters of dementia patients with distinct expression levels of the 40 analyzed genes, including IL-7Rα^low^ aging genes, which were associated with neurocognitive function as determined by MoCA, CDRsob and neuropsychological testing.

**Conclusions:**

We report differential expression of “normal” aging genes associated with IL‐7Rα^low^ EM CD8^+^ T cells in peripheral blood of patients with AD, and the significance of such gene expression in clustering subjects with dementia due to AD into groups with different levels of cognitive functioning. These results provide a platform for studies investigating the possible implications of age-related immune changes, including those associated with CD8^+^ T cells, in AD.

**Supplementary Information:**

The online version contains supplementary material available at 10.1186/s12979-023-00396-y.

## Background

Alzheimer’s disease (AD) is a chronic progressive neurodegenerative disorder and the most common cause of dementia, accounting for approximately 60–80% of all dementia cases [1]. Amyloid-β, a peptide derived from the amyloid precursor protein and known to assemble into extracellular amyloid plaques, is hypothesized to initiate AD pathogenesis by stimulating the formation of neurofibrillary tangles and contributing to the development of a cytotoxic environment prone to neurodegeneration [2]. Indeed, aducanumab and lecanemab, anti-amyloid antibody therapies, have been FDA-approved for treatment of mild cognitive impairment (MCI) and dementia due to AD. However, their therapeutic efficacy appears modest, with adverse effects [3–5], highlighting the need to investigate alternative pathogenic mechanisms and therapeutic targets for AD. Successive stressful events early in life including infections, ischemia, and metabolic insults are thought to result in a chronic, low-intensity inflammatory state that stimulates pathological Aβ accumulation and the development of the AD dementia syndrome [6, 7]. Previous studies have focused primarily on the innate immune system, including microglial cells which can be activated by Aβ, as a direct approach to evaluating neuroinflammation in the central nervous system (CNS) [8]. In contrast, fewer have investigated the potential role of adaptive immunity, including T cells, on AD pathology as part of a dysregulated systemic immune response resulting from harmful interactions between CNS immune cells and the peripheral immune system [9].

Aging is the strongest risk factor for AD [1]. In human T cells, probably the most prominent change with aging is the expansion of effector memory (CCR7^−^, EM) CD8^+^ T cells, which include CD45RA^+^ and CD45RA^–^ populations (note: hereafter EM indicates both CD45RA^+^ and ^–^ populations unless specified), in peripheral blood [10, 11]. One potential contributor to AD pathology includes cytotoxic and senescent T cell populations that may interact with the CNS through disruptions in the blood brain barrier [12, 13]. Previously, our lab found an age-associated expansion of highly inflammatory and cytotoxic EM CD8^+^ T cells expressing low levels of IL-7 receptor alpha chain (IL-7Rα or CD127)^low^, which have distinct characteristics including effector molecules, transcription factors, and DNA methylation profiles, in human peripheral blood [13–16] (also recently reviewed in reference [17]). Such T cell expansion, which is likely driven in part by repetitive immune stimulation over a lifetime [18], contributes to age-associated transcriptomic changes in human peripheral blood cells. About 15% of the age‐associated genes (231/1,497) reported by a meta‐analysis study of human peripheral blood from approximately 15,000 individuals corresponded to differentially expressed genes (DEGs) in IL-7Rα^low^ EM CD8^+^ T cells [19]. These genes, which are referred to as IL-7Rα^low^ aging genes, have been found to be associated with influenza vaccine responses in older adults [19], suggesting the possible biological implication of this gene signature in the development of immune responses, especially in the context of aging.

AD is heterogeneous and multifactorial in its etiologies, which limits any one AD model from being globally representative of disease pathogenesis and the mechanisms [20]. Evidence of systemic immune dysregulation in AD is growing, although a limited number of studies have investigated the role of adaptive immunity, including T cells, in AD [21]. Addressing the latter point is critical in that one of the most prominent changes with human aging is the expansion of memory CD8^+^ T cells with senescent characteristics in peripheral blood [11]. A recent study reported the expansion of CD45RA^+^ EM CD8^+^ T cells (T_EMRA_) in the cerebrospinal fluid of patients with dementia or MCI due to AD [22]. The clonal expansion of these cells in peripheral blood was found to also correlate with cognitive scores. Importantly, such cells are mostly IL-7Rα^low^ EM CD8^+^ T cells, implying the possible interface of such cell expansion with T cell immunosenescence in AD. Thus, the present study was conducted to determine whether an altered IL-7Rα^low^ EM CD8^+^ T cell associated aging gene signature (i.e., IL-7Rα^low^ aging genes) occurs in peripheral blood of individuals with AD compared to cognitively normal older adults (CN); especially in relation to genes identified to be possibly associated with AD by our systematic selection process of publicly available datasets. In addition, we aimed to assess the relationship of such alterations with disease severity as determined by in depth neurocognitive testing.

## Results

### A systematic search of publicly available data reveals a set of genes that are possibly altered in AD and belong to the IL-7Rα^low^ aging gene signature

**Fig. 1:**
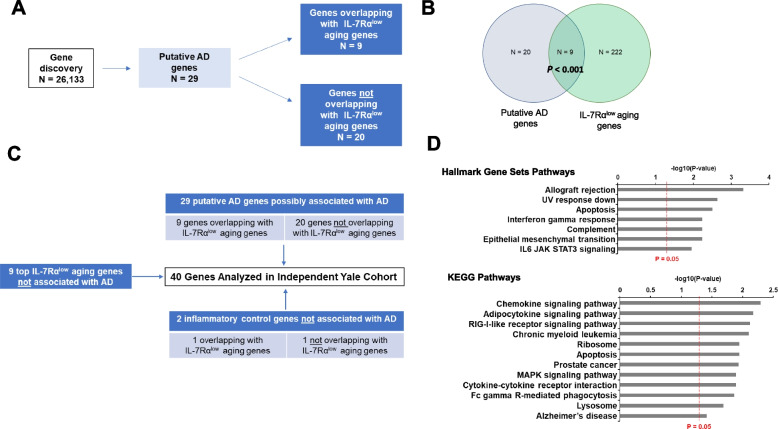
Gene discovery diagrams and Hallmark gene sets and KEGG pathways enriched by analyzed genes in independent Yale Cohort. **A**, **B** Gene discovery involved the analysis of 26,133 total genes based on publicly available databases that included AD (GSE140829, and GSE63060 and GSE63061 gene expression microarray datasets from a combined total of 428 patients with AD and 554 cognitively normal subjects, selected genes were those differentially expressed in ≥ 2 of these datasets) and memory (GSE127711) gene expression microarray datasets as well as the Molecular Signatures Database (MSigDB). This resulted in the discovery of 29 candidate genes (i.e., putative AD genes) which could be possibly altered in their expression levels in AD. Of the 29 genes, 9 were IL-7 receptor alpha low (IL-7Rα^low^) aging genes (*n* = 231). Gene discovery diagram **C** Showing the distinct characteristics of the analyzed gene categories. In addition to the genes identified by a systematic search of publicly available data, top IL-7Rα^low^ aging genes (i.e., IL-7Rα^low^ aging genes most associated with chronological age) were also studied. Hallmark gene sets and KEGG pathways **D** Identified by gene set enrichment analysis (GSEA) of the 40 analyzed genes

We explored whether any IL-7Rα^low^ aging genes (*n* = 231), which we reported in our published study [19], also belonged to a set of genes possibly associated with AD. To select the latter, we performed a systematic gene selection process on a large set of transcriptomic data derived from public domain gene set databases. We identified 3 publicly available peripheral blood gene expression microarray datasets (GSE140829, GSE63060 and GSE63061) from a combined total of 428 patients with AD and 554 cognitively normal subjects and selected a set of genes which were differentially expressed in ≥ 2 of these datasets [23, 24]. We also utilized a blood-based biomarker dataset (GSE127711) to identify genes of interest associated with short term memory dysfunction [25]. In addition, by searching the Molecular Signatures Database (MSigDB) [26] with keywords “Alzheimer” and “Alzheimer’s”, we identified several gene sets reportedly associated with AD to be included in our analysis (see Supplementary Methods 1.1 for more information). This systematic data search revealed a set of 29 genes of which expression could be possibly altered in AD (referred to as putative AD genes) (Fig. 1A). Of interest, 9 of the 29 putative AD genes (31%) were IL-7Rα^low^ aging genes (*P* < 0.001) (Fig. 1B) while the other 20 genes were not.

### IL-7Rα^low^ aging genes are differentially expressed in the peripheral blood of patients with AD

We next investigated whether the 29 putative AD genes, which were identified to be possibly associated with AD through our systematic approach above, had altered expression levels in peripheral blood of an independent cohort from the Yale Alzheimer’s Disease Research Center (ADRC) using RT-qPCR. We also analyzed 11 additional genes which were not associated with AD (i.e., no overlap with the 29 putative AD genes). These genes included 9 genes previously identified by our group to be differentially expressed in IL-7Rα^low^ EM CD8^+^ T cells and the most highly associated with chronological aging in healthy subjects as we previously reported (referred to as top IL-7Rα^low^ aging genes) as well as two inflammatory control genes (*IRF1* and *NLRX1*) which were not found in the AD datasets though they have the potential to affect AD pathology [19, 27]. The 9 top IL-7Rα^low^ aging genes were included to explore whether the expression levels of these genes, which were strongly associated with “normal” aging but altered in pathologic conditions such as HIV infection in older adults and frailty [28], changed in AD. In summary, the analyzed genes (*n* = 40) in our Yale cohort were 9 putative AD genes overlapping with IL-7Rα^low^ aging genes, 20 putative AD genes but not overlapping with IL-7Rα^low^ aging genes, 9 top IL-7Rα^low^ aging genes not overlapping with putative AD genes, and 2 inflammatory controls genes (Fig. 1C). These genes were found to be enriched in immune related pathways including chemokine signaling and cytokine-cytokine receptor interaction pathways in addition to AD (Fig. 1D). Indeed, these pathways have been suggested to be associated with AD though these genes have not yet been validated through more specific modalities of transcriptomic analyses such as RT-qPCR [29–32].

**Fig. 2:**
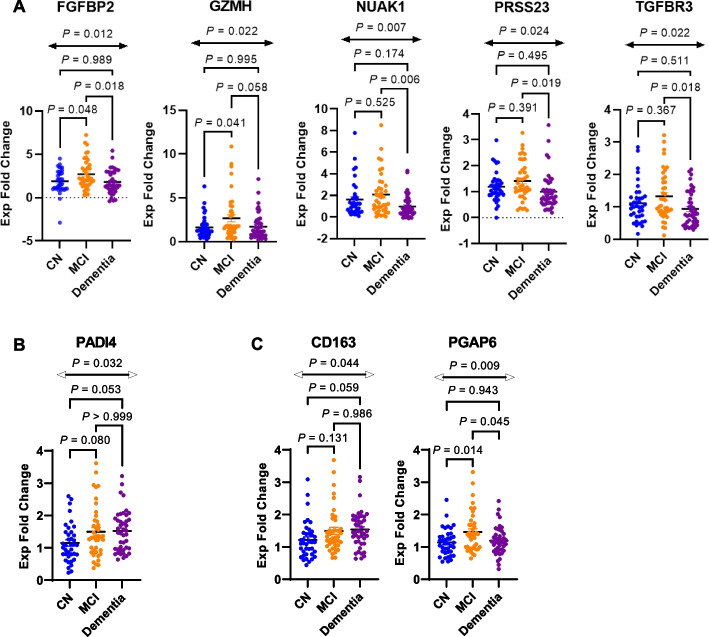
A set of genes associated with IL-7 receptor alpha (low) effector memory CD8^+^ T cells and Alzheimer’s disease (AD) is differentially expressed in peripheral blood of patients with AD. Reverse transcription qPCR (RT-qPCR) analysis showing differentially expressed genes in peripheral blood of cognitively normal (CN) and AD participants with mild cognitive impairment (MCI) or dementia. **A** Top IL-7 receptor alpha low (IL-7Rα^low^) effector memory (EM) CD8^+^ T cell-associated aging genes. **B** *PADI4,* an IL-7Rα^low^ aging gene found to be upregulated in 3 AD transcriptomic datasets. **C** Genes associated with AD transcriptomic datasets but not in the IL-7Rα^low^ EM CD8^+^ T cell associated aging gene signature. *P* values were obtained by ANOVA and adjusted during post-hoc multiple comparison testing using the Šidák correction

Our RT-qPCR analysis showed that eight genes (20%) out of the 40 analyzed were differentially expressed in the peripheral blood of the CN, MCI, and dementia groups (Fig. 2, Supplementary Figs. S1.1-S1.7. for all other gene expression plots). Six of the eight genes were IL-7Rα^low^ aging genes (75%, *P*-value = 0.120) [19], including *FGFBP2*, *GZMH*, *NUAK1*, *PRSS23*, *TGFBR3* and *PADI4* (Fig. 2A-B). Of these, five of the six genes were top IL-7Rα^low^ aging genes (62.5%, *P*-value = 0.002) (Fig. 2A) [19]. Except for *GZMH*, top IL-7Rα^low^ aging genes were found to be more highly expressed in the MCI group compared to the dementia group (*P*-value < 0.05), implying increased expression of top IL-7Rα^low^ aging genes in earlier stages of AD.

Of the genes associated with both IL-7Rα^low^ EM CD8^+^ T cells and AD (as per the 3 publicly available AD peripheral blood microarray datasets evaluated for this study), *PADI4* was found to be differentially expressed among the three clinical groups though adjusted *P*-values from post-hoc multiple comparison testing did not reach statistically significant levels between clinical groups (Fig. 2B). Two genes associated with AD and memory dysfunction [23–26] (but were not IL-7Rα^low^ aging genes) were also found to be differentially expressed (Fig. 2C). *CD163* encodes scavenger receptor cysteine-rich type 1 protein M130 which is expressed by monocytes and macrophages. This gene was found to be differentially expressed among the three clinical groups, though post-hoc multiple comparison testing suggests no significant differences between clinical groups. *PGAP6*, which encodes post-glycosylphosphatidylinositol attachment to proteins 6, was also found to be differentially expressed with higher expression levels in the MCI group compared to both the CN (*P*-value = 0.014) and dementia (*P*-value = 0.045) groups.

We performed GLM analysis to adjust for age, race, and sex. Four genes (*FGFBP2*, *PRSS23*, *TGFBR3*, *NUAK1*) out of the 5 top IL-7Rα^low^ aging genes remained differentially expressed with decreased relative gene expression in the dementia group compared to the MCI group (Supplementary Tables S3 and S4)*.* Further analysis of the genes differentially expressed in the public AD or memory datasets identified *PGAP6*, which is up-regulated in the MCI group compared to the dementia group (adjusted *P*-value = 0.029), and *AKAP13* (encodes A-kinase anchoring protein 13) which was differentially expressed between the CN and dementia groups (adjusted *P*-value = 0.023).

### Over-representation analysis (ORA) of DEGs demonstrates molecular and biological pathways associated with AD

**Fig. 3:**
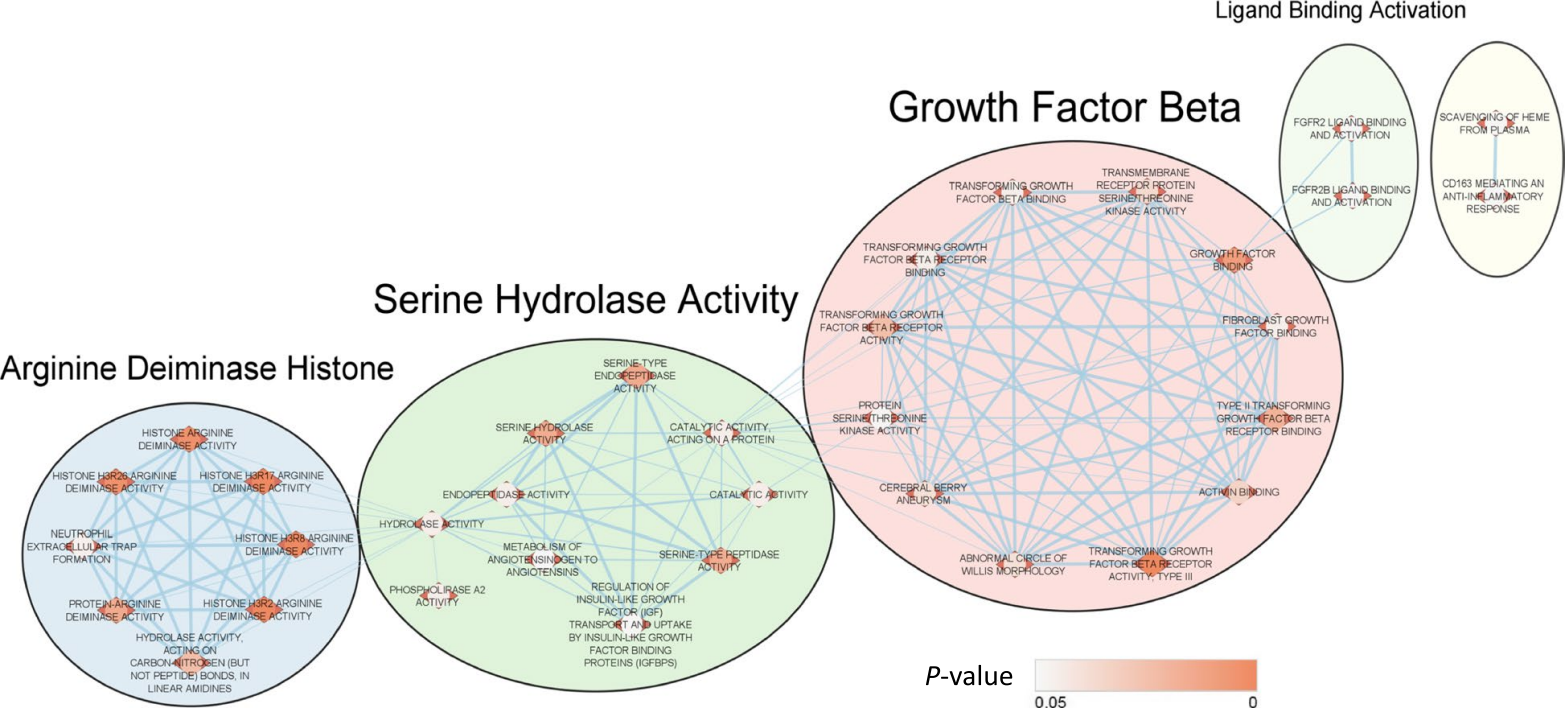
Differentially expressed genes (DEGs) are over-represented in pathways associated with Alzheimer’s disease (AD). Over-representation analysis (ORA) showing the 8 DEGs in Fig. 2 were over-represented in molecular and biological pathways thought to be linked to AD including TGF-β signaling, serine hydrolase activity, and histone modification. Auto-annotation descriptions of the 3 major clusters displayed corresponded with the pathways that were most significantly over-represented by the DEGs. Overrepresentation analysis significance was determined utilizing the Benjamini–Hochberg FDR multiple testing correction method by applying a significance threshold of 0.05. The pathways analyzed were obtained from readily available online databases including Gene Ontology (GO) pathways describing molecular function, cellular components, and biological processes; biological pathway databases including KEGG, Reactome, and WikiPathways; and the Human Phenotype Ontology database. Cytoscape applications were utilized to produce the displayed network map that organizes and automatically generates networks of overlapping gene sets that infer interactions between the identified pathways [37, 38]

To determine if the 8 DEGs discovered in our cohort through RT-qPCR analysis (Fig. 2) were also over-represented or more present in known biological and molecular interactions/pathways, ORA was conducted using the online program g:Profiler [33]. Over-representation of the DEGs in these pathways was compared to a background list of genes (see Supplementary File Background Gene List). The analysis identified multiple pathways that the 8 DEGs were highly over-represented. Such pathways included histone modification with arginine deiminase activity, serine-type hydrolase activity, and TGF-β signaling (Supplementary Table S5); all of which were previously associated with AD [34, 35] or have been suggested as potential pharmacologic targets [36]. Complementing the ORA findings listed in Supplementary Table S5, a word-based clustering algorithm showed that the pathways most significantly over-represented by the DEGs paralleled the 3 most common word descriptions annotating each pathway cluster as indicated by the annotations “Growth Factor Beta”, “Serine Hydrolase Activity”, and “Arginine Deiminase Histone” (Fig. 3). Additionally, edges (i.e., lines connecting nodes denoting associations between enriched pathways) generated between the 3 major clusters in the enrichment map imply potential functional, molecular, and biological interactions that could become robust targets of investigation for future studies of AD pathology.

### Neurocognitive function is associated with IL-7Rα^low^ aging gene expression in dementia due to AD

To uncover potential associations between aging genes and AD status, principal component analysis (PCA) was performed using Z-scores calculated from the expression levels of all 40 genes of interest (Supplementary Fig. S2). Minimal separation or clustering based on disease status was observed. Unbiased hierarchal clustering (Supplementary Fig. S3) was also performed, which demonstrated substantial portions of the MCI group and a small part of the dementia group clustered together with high expression levels of the top aging genes associated with IL-7Rα ^low^ EM CD8^+^ T cells (represented by the “top IL-7Rα^low^ aging genes” category in Supplementary Fig. S3).

**Fig. 4:**
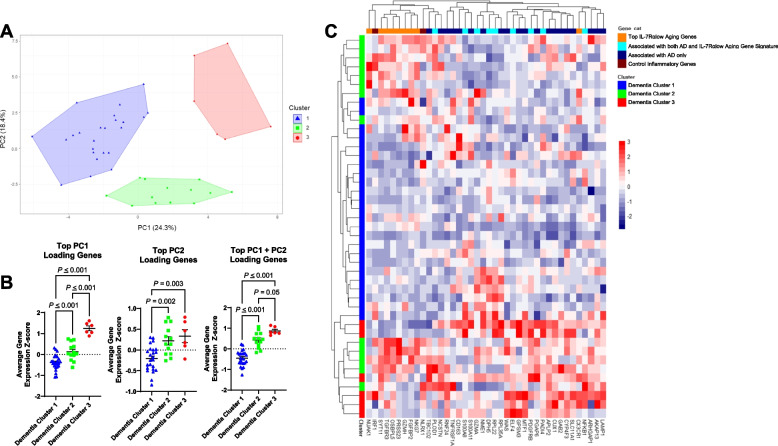
Unbiased clustering analyses of gene expression in the dementia group reveal three clusters of dementia patients with distinct levels of IL-7 receptor alpha (low) aging gene expression. **A** Principal component analysis (PCA) was done based on the expression levels of the 40 analyzed genes in peripheral blood of dementia participants. PCA yielded three clusters: Dementia Cluster 1 (blue triangles), Dementia Cluster 2 (green boxes), and Dementia Cluster 3 (red circles). **B** Average gene expression Z-scores of the top 10 loading genes for each principal component (PC) in the 3 clusters identified from (**A**) by PCA analysis of the dementia group. **C** Heatmap showing the results of unbiased hierarchal clustering analysis of gene expression in dementia participants. Subject clusters are based on PCA analysis in (**A**). *P* values were obtained by ANOVA with post-hoc multiple comparison testing or Welch’s *t*-test

We next performed PCA analysis in the dementia group to explore whether the 40 genes of interest, including IL-7Rα^low^ aging genes, could identify subsets of dementia subjects with different clinical characteristics (Supplementary Fig. S2). This analysis revealed three distinct clusters (subsets) within the dementia group (Fig. 4A). Notably, Cluster 1 dementia participants had lower average gene expression Z-scores of PC1 + PC2 top loading genes compared to both Clusters 2 and 3, suggesting similar gene expression patterns between the latter two clusters (Fig. 4B). Supporting this, unbiased hierarchal clustering (Fig. 4C) demonstrated a relative increase in gene expression levels of the top IL-7Rα^low^ aging genes in Clusters 2 and 3 compared to Cluster 1.

**Fig. 5:**
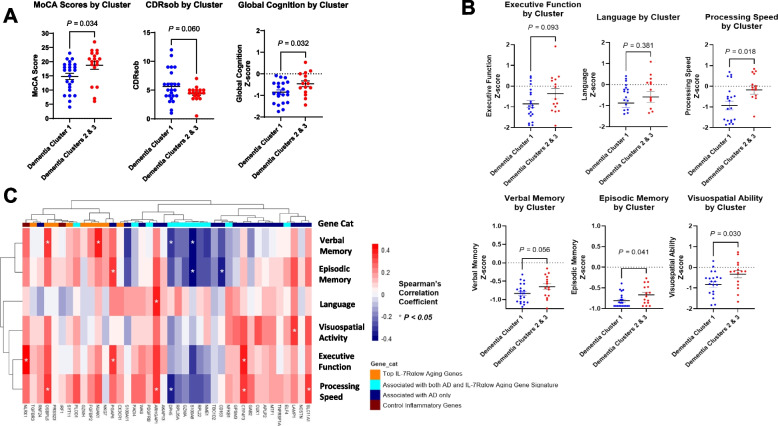
Clusters in dementia group associated with higher IL-7 receptor alpha (low) aging gene expression exhibit higher cognitive functioning according to comprehensive neuropsychological testing. **A** The Montreal Cognitive Assessment (MoCA) scores, Clinical Dementia Rating scale sum of boxes (CDRsob) scores, and Global Cognition composite scores of Dementia Cluster 1 compared to Dementia Clusters 2 & 3 combined. Dementia Clusters 1, 2 and 3 were identified based on PCA analysis in Fig. 4A. **B** Neuropsychological testing Z-scores of individual cognitive domains plotted according to dementia clusters. *P* values were obtained by unpaired *t*-test. **C** Unbiased hierarchal clustering heatmap of dementia participants’ Spearman’s rho coefficients (reflecting correlations between neuropsychological testing scores and all analyzed gene expression levels) were impartially categorized according to cognitive domain

To evaluate neurocognitive functioning based on the 3 clusters of participants in the dementia group, the Montreal Cognitive Assessment (MoCA) and Clinical Dementia Rating scale sum of boxes (CDRsob) scores (Supplementary Fig. S4) as well as neuropsychological testing Z-scores (Supplementary Figs. S5.1–5.6) were analyzed according to cluster designation. As Clusters 2 and 3 exhibited relatively similar gene expression profiles (Fig. 4B-C) and similar MoCA and CDRsob scores (Supplementary Fig. S4), both clusters were combined into one cluster which demonstrated significantly different MoCA scores (*P*-value = 0.034, Fig. 5A) and Global Cognition Z-scores (*P*-value = 0.032, Fig. 5A) compared to participants in Cluster 1. Furthermore, in comparing neuropsychological testing scores that reflect participants’ performance in several cognitive domains (i.e., episodic and verbal memory, executive function, processing speed, language, and visuospatial ability) [39], Cluster 1 participants had significantly lower neuropsychological testing scores compared to Clusters 2 and 3 in the domains of visuospatial ability, episodic memory, and processing speed (Fig. 5B). We evaluated the possible correlation of gene expression levels with neuropsychological testing scores as determined by Spearman correlation analysis. A set of genes, including *ARHGAP1 (CDC42GAP)*, *PGAP6, NUAK1, OSBPL5, CYP4F3, LAMP1*, *SLC11A1*, and *NLRX1*, showed a positive relationship with neuropsychological testing scores while *DPH5*, *S100A8* and *CD163* exhibited an inverse relationship with these measurements (Figs. 5C and S5.1.-S5.6). Additionally, unbiased hierarchal clustering of Spearman’s rho coefficients reflecting correlation of gene expression levels with neuropsychological testing scores revealed clustering of top IL-7Rα^low^ aging genes based on their correlation with individual cognitive domain scores (Fig. 5C). Executive function, processing speed, and verbal and episodic memory scores were found to most correlate with expression of all genes analyzed (Fig. 5C).

## Discussion

A substantial body of evidence supports the occurrence of immune dysregulation in AD, affecting both central and peripheral immune responses [21]. Advanced age, the strongest risk factor for AD, is known to induce alterations in the immune system, a phenomenon referred to as immunosenescence [11, 40, 41]. This raises the possibility of an interface between immunosenescence and AD. Declining immunity can account in part for increased risks of infections and malignancies in older adults. Another process called “inflammaging”, which is characterized by elevated levels of circulating pro-inflammatory molecules such as IL-6, also occurs with age [42]. A meta-analysis of studies measuring peripheral blood cytokines found increased levels of IL-6, TNF-α, and IL-1β, primarily produced by innate immune cells, in AD subjects compared to control subjects [43]. Also, chronic low-grade inflammation defined by increased C-reactive protein (CRP), an acute phase reactant induced by IL-6 and IL-1β, was associated with shortened latency for onset of AD in individuals carrying the *apolipoprotein E4 (ApoE4)* allele in a population-based cohort study [44]. These observations support the possible interaction between innate immune-driven inflammaging and the development of AD. However, it is largely unknown whether a similar interaction occurs between AD and age-associated adaptive immune changes. In adaptive immunity, one of the most notable alterations with age is the expansion of T cells, especially EM CD8^+^ T cells [10, 11]. We previously found an age-associated expansion of highly inflammatory and cytotoxic IL-7Rα^low^ EM CD8^+^ T cells [13–17], which contributes to the peripheral blood transcriptomic changes with age through IL-7Rα^low^ EM CD8^+^ T cell associated aging gene signature (i.e., IL-7Rα^low^ aging genes) [19]. Of note, a recent study reported the expansion of CD45RA^+^ EM CD8^+^ T cells, which were largely IL-7Rα^low^, in the cerebrospinal fluid and peripheral blood of patients with AD [22], suggesting a possible interface between AD immune dysregulation and T cell immunosenescence. Indeed, our study bolsters this notion by demonstrating that the expression levels of a group of genes, including IL-7Rα^low^ aging genes and those identified as differentially expressed in AD by global transcriptomics studies, are altered in peripheral blood of AD subjects compared to CN subjects. Further, we found these gene expression patterns are also associated with neurocognitive function in AD subjects with dementia.

Our systematic analysis of publicly available data, including those from global transcriptomic analyses, revealed 29 putative AD genes which could be possibly associated with AD. Nine of the 29 putative AD genes (31%) were aging genes enriched in IL-7Rα^low^ EM CD8^+^ T cells, suggesting the possible relationship of this cell subset with AD. We attempted to validate these findings by analyzing expression levels of these 29 genes and 9 additional top IL-7Rα^low^ aging genes in peripheral blood of CN and patients with AD using RT-qPCR. The results of our RT-qPCR analysis showed 8 DEGs between AD and CN groups. Of note, six (75%) of the 8 genes were IL-7Rα^low^ aging genes; five of which were top IL-7Rα^low^ aging genes. These “top IL-7Rα^low^ aging genes” have been previously associated with IL‐7Rα^low^ EM CD8^+^ T cells [19], a T cell population characterized by expressing senescent markers that expand with age, although any expressional changes of these genes in AD were not previously reported. These top IL-7Rα^low^ aging genes include genes related to cytotoxic molecules (*FGFBP2, GZMH, PRSS23*) as well as pathways that have been associated with cell survival and cell cycle regulation (*PRSS23*, *NUAK1*) [40, 41]. These genes have been previously associated with tumor growth and metastasis (*NUAK1*, *TGFBR3*, *FGFBP2*) [45, 46], as well as autoimmune disorders (*PRSS23*) [47]. In relation to AD, *NUAK1* overexpression is suspected to promote tau hyperphosphorylation based on findings in AD mouse models [48]. Interestingly, these “top IL-7Rα^low^ aging genes”, which were found in healthy non-AD individuals, appeared more highly expressed in the MCI group compared to the CN and dementia groups. Similar patterns have been observed in a previous study which found increases in myeloid-derived suppressor cells and FOXP3^+^ CD4^+^ regulatory T cells in the peripheral blood of MCI participants compared to age-matched healthy and mild dementia participants [49]. This pattern could be reflective of increasing immune activity and an initial anti-inflammatory response early in the disease course before the shift to full suppression of inflammation found in neurodegeneration. Also, a decrease in the expression of “top IL-7Rα^low^ aging genes” was noticed in peripheral blood of frail older adults compared to non-frail older adults [28], suggesting a potential impairment in the development of a normal aging gene signature in pathologic conditions such as dementia and frailty in older adults. These points can be further explored by a future study that prospectively analyzes the expression levels of IL-7Rα^low^ aging genes and examines their relationship with disease progress in AD.

ORA revealed that the 8 DEGs were also highly present (over-represented) in the biological and molecular pathways of arginine deiminase activity, serine-type hydrolase activity, and TGF-β signaling which could be related to AD pathology. For example, peptidyl arginine deiminase was previously found to co-localize with amyloid beta-42 in the hippocampus and is postulated to mediate enhancement of histone modification and citrullination, thus promoting neutrophil extracellular trap formation which would contribute to the pro-inflammatory milieu in AD [50, 51]. This is notable as *PADI4*, which encodes the only peptidyl arginine deiminase enzyme that translocates into the nucleus, tended to be more highly expressed in AD participants compared to CN older adults implying an epigenetic effect being observed in our cohort. Inhibition of acetylcholinesterase, a metabolic serine hydrolase, has been a mainstay of treating cognitive symptoms in patients with AD [36]. Lastly, dysfunctional TGF-β signaling has been reported as a possible cause of amyloid beta accumulation and neurodegeneration [52].

Regarding associating differential expression with the clinical phenotypes of AD in our study, subgroup analyses of the clinical groups revealed distinct clustering and separation of dementia participants in PCA and unbiased hierarchal clustering. Dementia participants that were separated into Clusters 2 and 3 were noted to exhibit higher gene expression levels of IL‐7Rα^low^ aging genes compared to Cluster 1 participants. Interestingly, Clusters 2 and 3 dementia participants also performed better during neuropsychological testing compared to those in Cluster 1. These findings suggest that IL-7Rα^low^ aging signature originally found in normal non-AD individuals may decline with the progress of dementia in AD. Indeed, this notion is also supported by our finding of decreased expression levels of “top IL-7Rα^low^ aging genes” in dementia subjects compared to MCI subjects.

We evaluated the possible correlation of gene expression levels with neuropsychological testing scores in dementia subjects. Genes, including *ARHGAP1 (CDC42GAP)*, *LAMP1*, *NLRX1*, *DPH5*, *S100A8* and *CD163*, with significant levels of correlation between expression levels and neuropsychological testing scores could also be possibly implicated in the development of AD. Mice with a heterozygous deficiency of *Cdc42GAP* that encodes CDC42 GTPase-activating protein had impaired cognitive behavior, neuronal senescence, and the pathological phenotypes of Alzheimer's disease [53]. Lysosomal association membrane protein 1, encoded by the gene *LAMP1*, was reported to ameliorate the inflammatory response in AD animal models by enhancing autolysomal function which could be altered in AD [54]. The loss of NLRX1, which can negatively regulate NF-kB signaling, was found to exacerbate neural tissue damage in an animal model of brain injury [55]. In our study, the expression levels of these genes, including *ARHGAP1 (CDC42GAP)*, *LAMP1*, and *NLRX1*, had a positive relationship with neurocognitive functioning scores. In contrast, the expression levels of *S100A8*, *DPH5* and *CD163* had an inverse relationship with neuropsychological testing scores. S100A8 (calgranulin A), a protein that forms the inflammatory molecule calprotectin with S100A9, was found to increase in the sera of AD subjects [56] and in the hippocampus of AD mice [57]. From a functional perspective, S100A8/A9 (calprotectin) can enhance leukocyte recruitment and cytokine production [58]. Additionally, aggregated Aβ induced expression of *S100A8* mRNA was found in human microglial cells, suggesting the local production of this inflammatory molecule in the brain of AD [59]. Of note, S100A8/A9 (calprotectin) has been considered as a biomarker for inflammatory diseases such as inflammatory bowel disease [58, 60]. Also, increased expression of CD163, a membrane-bound scavenger receptor expressed on perivascular macrophages, was found in the parenchymal microglia of AD patients [61]. A combination of these findings with the results of our study raises the possibility of future studies investigating the clinical significance of these genes and/or proteins encoded by the same genes in AD.

It is noteworthy that six of the eight DEGs identified through qPCR analysis of the 40 genes in our dementia, MCI, and CN groups were IL-7Rα^low^ aging genes. In contrast, only 2 genes, including *CD163* and *PGAP6*, of the 20 putative AD genes not overlapping with IL-7Rα^low^ aging genes which were identified from global transcriptomics data showed differential expression levels in the same cohort. These findings imply that AD is accompanied by an impairment in the development normal aging gene signature, such as IL-7Rα^low^ aging genes. Critically, the expression levels of six of these 20 putative AD genes (*PGAP6*, *ARHGAP1, CD163, CYP4F3, LAMP1, SLC11A1)* correlated with neuropsychological testing scores in the dementia subjects, suggesting that the genes associated with disease severity of dementia can be different from those with altered expression levels in AD as compared to CN subjects. Indeed, our PCA analysis showed that the gene set of our study, consisting of 40 genes including IL-7Rα^low^ aging genes and putative AD genes from global transcriptomics studies, could separate dementia subjects into clusters with distinct neuropsychological testing scores. However, PCA analysis based on the same gene set could not distinguish between the dementia, MIC, and CN groups. This point suggests that some biomarkers are informative in identifying dementia subjects with distinct neurocognitive function only within the group of dementia subjects but perhaps not in discerning them from CN and MCI subjects.

There are several limitations of this study. Participants were matched according to chronological age to minimize confounding due to this variable although there are other potential confounding variables (*e.g.,* biological aging) that we are unable to control. Additionally, the low participation rates of under-represented peoples in the MCI and dementia groups could be a limitation of our study. Considering the heterogeneity of immune cells, including EM CD8^+^ T cells, in peripheral blood, transcriptomics analysis at the single cell level can be conducted to investigate the relationship of our findings with the possible changes in circulatory immune cell heterogeneity in AD. Lastly, further studies are warranted to determine if peripheral blood gene expression is reflective of immune alterations found in the CNS.

## Conclusion

Altogether, the gene expression patterns in this AD cohort suggest an altered immune response compared to healthy normal aging. Crucially, there is differential gene expression of normal aging genes associated with IL‐7Rα^low^ EM CD8^+^ T cells in AD, and the expression patterns of such genes, along with AD-associated genes, could distinguish dementia subjects with different levels of neurocognitive function. As AD progresses, subjects may lose normal aging-related immune responses while developing AD-related immune responses. The gene expression patterns found in our study could be utilized to develop predictive models for disease trajectories earlier in the course of AD. Taken all together, our findings allude to the potential impact of peripheral immune alterations, including those associated with aging, in contributing to neuroinflammatory processes associated with AD.

## Materials and methods

Additional detailed methods are provided in Supplementary Methods.

### Participant characteristics and neuropsychological evaluation

A total of 121 whole blood samples from participants recruited by the Yale ADRC were obtained. These include 38 CN subjects, 40 subjects with MCI due to AD, and 43 with dementia due to AD (see Table 1 for demographic characteristics). MCI and probable dementia due to AD were defined according to the National Institute on Aging – Alzheimer’s Association (NIA-AA) guidelines [62, 63]. Clinical data from each participant, including biomarkers and cognitive testing scores, were utilized to produce a consensus diagnosis based on review by a multidisciplinary panel of experts from the Yale ADRC. Severity and staging were assessed using clinical rating scales including the Montreal Cognitive Assessment (MoCA) [64], and Washington University’s Clinical Dementia Rating (CDR) scale [65]. Cognitive functioning was also evaluated utilizing the neuropsychological testing battery from the National Alzheimer’s Coordinating Center’s Uniform Data Set. The scores from this comprehensive cognitive testing were used to generate composite Z-scores for cognitive domains including episodic and verbal memory, executive function, processing speed, language, and visuospatial ability [39]. These Z-scores were averaged for each participant to generate a “Global Cognition” Z-score to represent the overall cognitive functioning of each participant based on the neuropsychological testing results.

**Table 1:** Demographics of study participants

	Cognitively Normal (CN) (*N* = 38)	Mild Cognitive Impairment (MCI) (*N* = 40)	Dementia (*N* = 43)	*P* -value
Age	72.3 ± 8.6	72.7 ± 7.4	71.2 ± 8.2	0.671
Female/Male	23 (60.5%) / 15 (39.5%)	17 (42.5%) / 23 (57.5%)	22 (51.2%) / 21 (48.8%)	0.282
Race				
Non-Hispanic White	22 (57.9%)	31 (77.5%)	40 (93.1%)	0.009
Black	10 (26.3%)	4 (10%)	1 (2.3%)	
American Indian or Native Alaskan	2 (5.3%)	0	1 (2.3%)	
Asian	1 (2.6%)	1 (2.5%)	0	
Unknown	3 (7.9%)	4 (10%)	1 (2.3%)	
Cognitive Scores				
MoCA	25.7 ± 3.2	20.3 ± 4.9	16.1 ± 6.3	< 0.001
CDRsob	0.2 ± 0.5	2.2 ± 1.0	5.1 ± 2.3	< 0.001

Demographic characteristics of 121 Alzheimer’s Disease Research Center (ADRC) participants involved in this study and organized according to clinical group. *P*-values were obtained by the ANOVA or Chi-square test

*Abbreviations*: *MoCA* Montreal Cognitive Assessment, *CDRsob* Clinical Dementia Rating sum of boxes

### Quantitative Polymerase Chain Reaction (qPCR)

RNA was isolated from blood stored in heparinized tubes at -80˚C using a modified QIAGEN RNeasy Kit protocol (see Supplementary Methods 1.2). Following cDNA synthesis, target gene expression was measured by qPCR (see supplementary methods for details including data processing).

### Statistical analyses

One-way ANOVA and Pearson’s chi-squared tests were for descriptive data analysis. General linear models (GLM) were also used to analyze differences in relative gene expression levels utilizing estimated least-square means generated after adjusting for age, sex, and race. Fisher’s exact test was performed to calculate significance of overlap between 40 genes and gene sets. Spearman’s rho coefficients were calculated to determine if there were any significant correlations between neuropsychological testing scores and gene expression.

### Data sharing and code availability statement

This study included analysis of transcriptomic microarray data from publicly available datasets (GSE140829, GSE127711, GSE63060 and GSE63061) which are available from the Gene Expression Omnibus. Additional de-identified data utilized in this manuscript can be provided upon request from the Yale ADRC.

### Study approval

The Yale University Institutional Review Board approved the collection and processing of all whole blood samples investigated in this study (IRB protocol ID 2000028709). Informed consent was obtained from all participants at the time of collection by ADRC staff.

### Supplementary Information

**Additional file 1:** **Supplementary Methods.** 1.1. Gene Discovery. 1.2. RNA Isolation and Complementary DNA synthesis Protocol. 1.3. Quantitative Polymerase Chain Reaction. 1.4. Data Processing. 1.5. Statistical Analyses. **Table S1.** Gene Targets’ Primer Sequences. **Table S2.** Gene Targets Analyzed. **Table S3.** GLM analysis for differentially expressed genes. **Table S4.** LSM differences between clinical groups generated from GLM analysis for differentially expressed genes. **Table S5.** Molecular and biological pathways over-represented by differentially expressed genes. **Figure S1.1.** Top IL-7Rα^low^ aging genes. **Figure S1.2.** IL-7Rα^low^ aging genes downregulated in at least 2 AD datasets and were not associated with Memory Dataset. **Figure S1.3.** IL-7Rα^low^ aging genes associated with AD per MsigDB and upregulated in at least 2 AD datasets. **Figure S1.4.** IL-7Rα^low^ aging genes associated with Memory Dataset and AD per MsigDB and/or upregulated in at least 2 AD Datasets. **Figure S1.5.** AD genes associated with memory database, not IL-7Rα^low^ aging genes. **Figure S1.6.** Genes and downregulated in 3 AD datasets; not IL-7Rα^low^ aging genes. **Figure S1.7.** Inflammatory control genes. **Figure S2.** All Genes PCA Plot. **Figure S3.** Hierarchal Clustering Heatmap. **Figure S4.** MoCA and CDRsob Scores per Dementia Cluster. **Figure S5.1.** Processing Speed Z-scores vs. Gene Expression Z-scores. **Figure S5.2.** Verbal Memory Z-scores vs. Gene Expression Z-scores. **Figure S5.3.** Episodic Memory Z-scores vs. Gene Expression Z-scores. **Figure S5.4.** Executive Function Z-scores vs. Gene Expression Z-scores. **Figure S5.5.** Language Z-scores vs. Gene Expression Z-scores. **Figure S5.6.** Visuospatial Ability Z-scores vs. Gene Expression Z-scores.

**Additional file 2.** Background gene list.

## Data Availability

This study included analysis of transcriptomic microarray data from publicly available datasets (GSE140829, GSE127711, GSE63060 and GSE63061) which are available from the Gene Expression Omnibus. Additional de-identified data utilized in this manuscript can be provided upon request from the Yale ADRC.

## Abbreviations

AD: Alzheimer’s disease
CDRsob: Clinical Dementia Rating scale sum of boxes
CN: Cognitively normal persons
CNS: Central nervous system
DEGs: Differentially expressed genes
EM: Effector memory
GLM: General linear models
IL-7Rα^low^: IL-7 receptor alpha low
MCI: Mild cognitive impairment
MoCA: Montreal Cognitive Assessment
ORA: Over-representation analysis
PCA: Principal component analysis
