# Supplementary material for: Aging gene signature of memory CD8+ T cells is associated with neurocognitive functioning in Alzheimer’s disease
Source: Immun Ageing. 2023 Dec 2;20:71. doi: 10.1186/s12979-023-00396-y (PMC10693128; doi:10.1186/s12979-023-00396-y)
Supplement: Supplementary file 1 — Additional file 1: Supplementary Methods. 1.1. Gene Discovery. 1.2. RNA Isolation and Complementary DNA synthesis Protocol. 1.3. Quantitative Polymerase Chain Reaction. 1.4. Data Processing. 1.5. Statistical Analyses. Table S1. Gene Targets’ Primer Sequences. Table S2. Gene Targets Analyzed. Table S3. GLM analysis for differentially expressed genes. Table S4. LSM differences between clinical groups generated from GLM analysis for differentially expressed genes. Table S5. Molecular and biological pathways over-represented by differentially expressed genes. Figure S1.1. Top IL-7Rαlow aging genes. Figure S1.2. IL-7Rαlow aging genes downregulated in at least 2 AD datasets and were not associated with Memory Dataset. Figure S1.3. IL-7Rαlow aging genes associated with AD per MsigDB and upregulated in at least 2 AD datasets. Figure S1.4. IL-7Rαlow aging genes associated with Memory Dataset and AD per MsigDB and/or upregulated in at least 2 AD Datasets. Figure S1.5. AD genes associated with memory database, not IL-7Rαlow aging genes. Figure S1.6. Genes and downregulated in 3 AD datasets; not IL-7Rαlow aging genes. Figure S1.7. Inflammatory control genes. Figure S2. All Genes PCA Plot. Figure S3. Hierarchal Clustering Heatmap. Figure S4. MoCA and CDRsob Scores per Dementia Cluster. Figure S5.1. Processing Speed Z-scores vs. Gene Expression Z-scores. Figure S5.2. Verbal Memory Z-scores vs. Gene Expression Z-scores. Figure S5.3. Episodic Memory Z-scores vs. Gene Expression Z-scores. Figure S5.4. Executive Function Z-scores vs. Gene Expression Z-scores. Figure S5.5. Language Z-scores vs. Gene Expression Z-scores. Figure S5.6. Visuospatial Ability Z-scores vs. Gene Expression Z-scores. [file 12979_2023_396_MOESM1_ESM.docx]

**Supplementary Materials**

1. **Supplementary Methods**
2. **Supplementary Tables**
3. **Supplementary Figures**

**1. Supplementary Methods**

- 1. **Gene Discovery**

Forty genes of interest were identified from publicly available databases based on their associations with at least one transcriptomic dataset or their relation to significant inflammatory or aging markers. Datasets that were utilized in gene discovery include microarray expression profiles (GSE140829.GPL15988 (1) and GSE63063.GPL6947 and GLPL10558 (2)) derived from total RNA obtained from the peripheral blood of dementia patients. Additional genes were obtained from the molecular signature database (MSigDB) (3) which included over 6,700 gene sets originating from a diverse set of sources and tissue types. Specifically, data were obtained from tissue and microarray databases associated with Alzheimer’s disease (AD) pathways (4), as well as direct brain tissue and endothelial cell microarray data assorted by Blalock et al. (5) and Wu et al. (6). Lastly, a blood-based biomarker database geared towards early detection of AD risk was utilized to discover genes of interest that were associated with short memory dysfunction as measured by the Hopkins Verbal Learning Test (7). After discovery, these genes were separated into the following categories: (A) Genes associated with both AD and the IL-7Rα^low^ Aging Gene Signature, (B) AD associated genes not found in the IL-7Rα^low^ Aging Gene Signature, (C) top IL-7Rα^low^ Aging Genes (8), (D) Control genes associated with aging but not specifically AD or IL-7Rα^low^ Aging Genes.

- 1. **RNA Isolation and Complementary DNA synthesis Protocol**

RNA was isolated using a modified QIAGEN RNeasy Kit protocol. Frozen whole blood was allowed to thaw in QIAzol Lysis Reagent (QIAGEN) and homogenized by pipetting. 1 mL of homogenate was transferred into a 1-2mL microcentrifuge tube where 200 µl of chloroform was added and the mixture shaken vigorously for 15 seconds. Afterwards, the mixture was incubated at room temperature for 2-3 minutes and then centrifuged at 10,000 RPMs (Eppendorf 5417R Centrifuge) for 15 minutes at 4˚C. After centrifugation, 400-500 µl of the upper aqueous phase of the mixture was transferred to a new microcentrifuge tube taking care to avoid the interphase. 70% ethanol was added at a 1:1 volume ratio and the mixture was vortexed. 700 µl of the resulting mixture was then transferred to a RNeasy Mini spin column (QIAGEN) and centrifuged at room temperature for 15 s at ≥8000 x g. The flow-through was discarded and repeated passing of the ethanol-sample mixture through the RNeasy Mini spin column was done until none of the original mixture was left. The DNase Digest protocol (QIAGEN) was then conducted to purify the sample RNA. Afterwards, the sample RNA was washed with 500 µl Buffer RPE two times and dried by centrifuging at full speed (≥ 13,000 RPM) for 3-5 min. The RNeasy Mini spin column was placed in a new microcentrifuge tube and 30 µl of RNase-free water was added and left to rest for 1 minute before centrifuging for 1 min at ≥ 10,000 RPM to collect the resulting RNA solution. The RNA solution was placed in an ice bath and was subsequently tested for quality and quantity via the NanoDrop™ 2000 Spectrophotometer. RNA templates of good quality and quantity were then utilized for complimentary DNA (cDNA) synthesis utilizing the iScript cDNA synthesis kit protocol (Bio-Rad).

**1.3 Quantitative Polymerase Chain Reaction**

Target gene expression was measured by quantitative polymerase chain reaction (qPCR) analysis by producing 10 µl reaction mixtures containing cDNA, SYBR Green Supermix (Bio-Rad) and target gene primers at 1 µM concentrations (see Supplementary Table S1 for target gene primer RNA nucleotide sequences). One 384-well plate (Bio-Rad) was used for qPCR analysis of each analyzed gene to measure gene expression of all samples in one qPCR experiment in order to avoid experimental batch effects between clinical groups. β-actin was utilized as the housekeeping gene for all qPCR experiments. The reaction mixture was initially denatured at 95˚C for 3 minutes and then underwent 40 cycles of the following: denatured for 15 seconds at 95˚C then annealing, extension, and read fluorescence for 45 seconds at 60˚C using the CFX384 Touch Real-Time PCR Detection System (Bio-Rad). The expression levels for each gene were then calculated per the 2^-ΔΔC^_T_ equation (9).

**1.4 Data Processing**

Approximately 2.6% of all expression fold change values comprising the current transcriptomic dataset were considered missing completely at random. Using Bioconductor’s “pcaMethods” software, missing values were imputed utilizing probabilistic principal component analysis (PPCA), a method of multiple imputation based on a probabilistic model created via a maximum likelihood estimation approach (10, 11). ComBat, a program in the Bioconductor’s “sva” software suite that utilizes empirical Bayes regression to adjust for and correct uncontrollable batch effects (12,13) was used to process the transcriptomic dataset generated from the RT-qPCR analysis.

*Power calculation.* To estimate the minimum sample size needed to demonstrate differential gene expression between CN and AD participants at a significance level of 0.05, power calculations were done (with an assumed power of 0.8) utilizing preliminary gene expression data of the first 60 participants included in this study.

**1.5 Statistical Analyses.**

One-way ANOVA and Pearson’s chi-squared tests were performed as part of a descriptive analysis of ADRC participant demographic characteristics of age and sex, respectively. Due to the limited participation of non-White persons, analyses adjusting for race were conducted by labeling participants as either “non-Hispanic White” or “Other” with the latter group comprised of Hispanic and non-White participants. One-way ANOVA testing was completed to determine if there were differences in mean gene expression among the three clinical groups. General linear models (GLM) were also used to analyze differences in relative gene expression levels utilizing estimated least-square means (LSM) generated after adjusting for age, sex, and race. Fisher’s exact test was performed to calculate significance of overlap between 40 genes and gene sets. ORA was performed using g:Profiler (version e108_eg55_p17_0254fbf) (14), and the following Cytoscape (version 3.9.1) (15) applications: EnrichmentMap version 3.35 (16), WordCloud version 3.1.4 (17), and AutoAnnotate version 1.4.0 (18). This was conducted using the Jaccard Overlap Combined Index test (k constant = 0.5) with an “Edge cutoff” of 0.62, *P*-value of 0.05, and q-value of 0.1. Welch’s *t*-test was conducted to determine if there were differences in MoCA scores, CDRsob scores, and Global Cognition Z-scores between dementia clusters. Unpaired *t*-tests were conducted to show any differences in neuropsychological testing cognitive domain Z-scores between dementia clusters. Spearman’s rho coefficients were calculated to determine if there were any significant correlations between neuropsychological testing scores and gene expression. All analyses were conducted to test for statistical significance in a “two-tailed” manner when applicable. Data was processed and analyzed using IBM SPSS Statistics for Windows, version 28.0, released in 2021, Armonk, NY: IBMCorp, R version 4.2.2, and GraphPad Prism version 9.5.0 for Windows, GraphPad Software, San Diego, California USA.

We utilized publicly available R (version 4.2.2) packages and programs Bioconductor: “pcaMethods” ver. 1.90.0 (10,11), “ComBat” from “sva” package ver. 3.46.0 (12,13), “BatchQC” ver. 1.26.0 (19), “limma” ver. 3.54.1 (20); Miscellaneous R packages/programs: “corrplot” ver. 0.92 (21), “FactoMineR” ver. 2.7 (22), “factoextra” ver. 1.0.7 , “ggbiplot” ver. 0.55 (23), “pheatmap” ver. 1.0.12 (24), “tidyverse” ver. 2.0.0 (25), “cluster” ver. 2.1.4 (26) for statistical analyses and generating plots/figures.

**Supplementary Tables**

**Table S1. Gene Targets’ Primer**  **Sequences**

**Table S2. Gene Targets Analyzed**


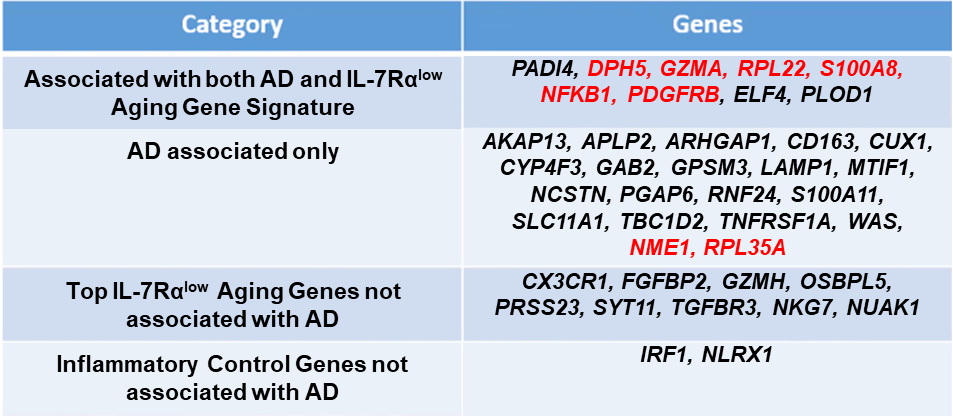


**Table S3. GLM analysis for differentially expressed genes**

Table demonstrating F statistics and corresponding *P*-values calculated from GLMs for differentially expressed genes. GLMs were adjusted for age, sex and race, and standard error (95% CI) by clinical group. Four out of the nine top IL-7Rα^low^ aging gene targets (*FGFBP2*, *PRSS23*, *TGFBR3*, *NUAK1*) that were analyzed by qPCR remained differentially expressed at a significance level of 0.05. Abbreviations: GLM, general linear model

**Table S4. LSM differences between clinical groups generated from GLM analysis for differentially expressed genes**

Table displaying post-hoc multiple comparison testing adjusted *P-*values after Šidák correction for differentially expressed genes suggesting lower levels of expression in the dementia group compared to the MCI group for most aging genes after adjustment (lower LSM estimations reflective of lower predicted relative gene expression). Abbreviations: GLM, General linear model; LSM, least-squares means; CN, cognitively normal; MCI, mild cognitive impairment

**Table S5. Molecular and biological pathways over-represented by differentially expressed genes**

**Supplementary Figures**

**Figure S1.1. Top IL-7Rα^low^ aging genes**

**Figure S1.2. IL-7Rα^low^ aging** **genes downregulated in at least 2 AD datasets and were not associated with Memory Dataset**

**Figure S1.3. IL-7Rα^low^ aging** **genes associated with AD per MsigDB and upregulated in at least 2 AD datasets**

**
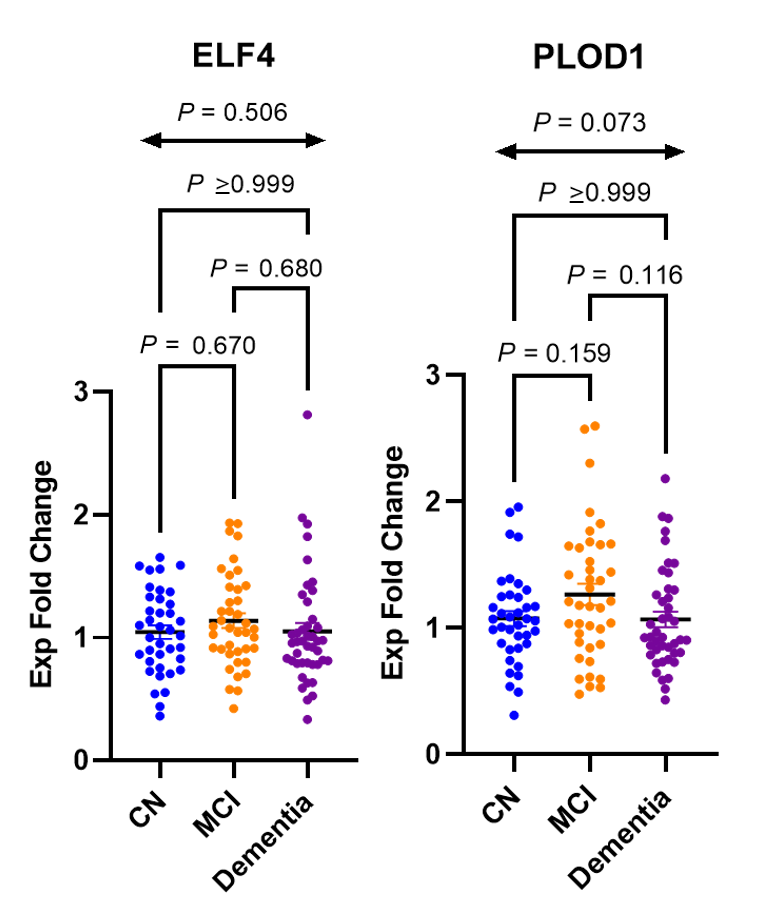
**

**Figure S1.4. IL-7Rα^low^ aging** **genes associated with Memory Dataset and AD per MsigDB and/or upregulated in at least 2 AD Datasets**

**
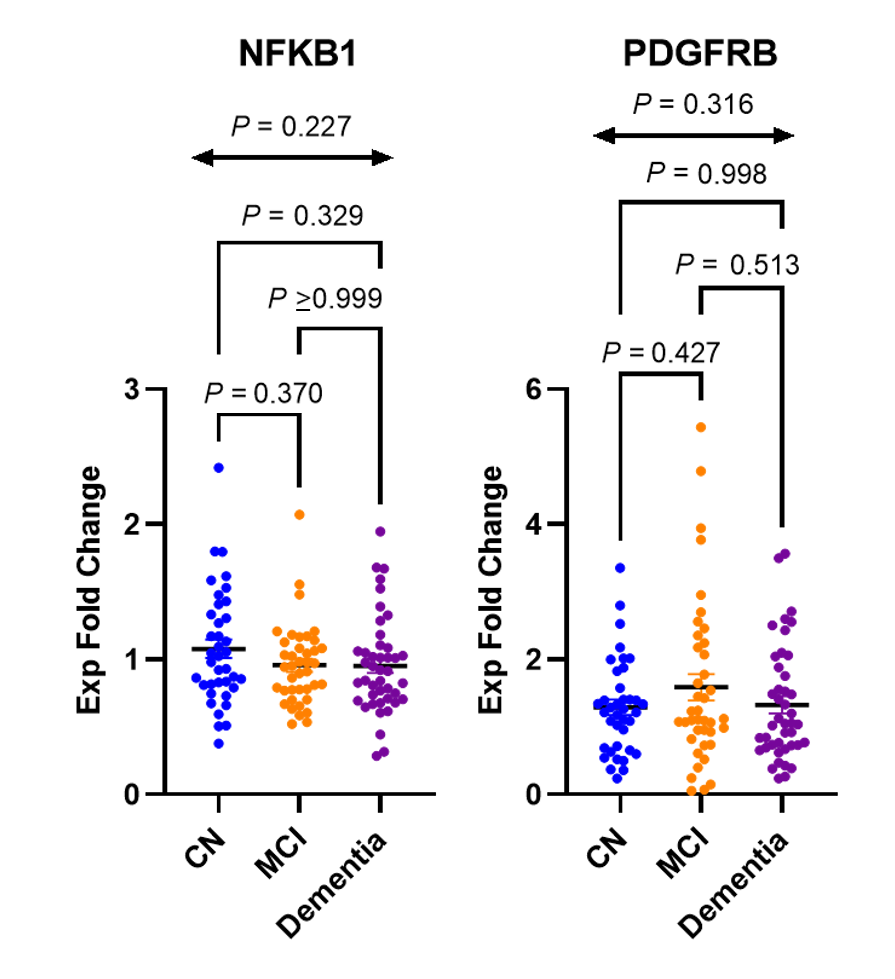
**

**
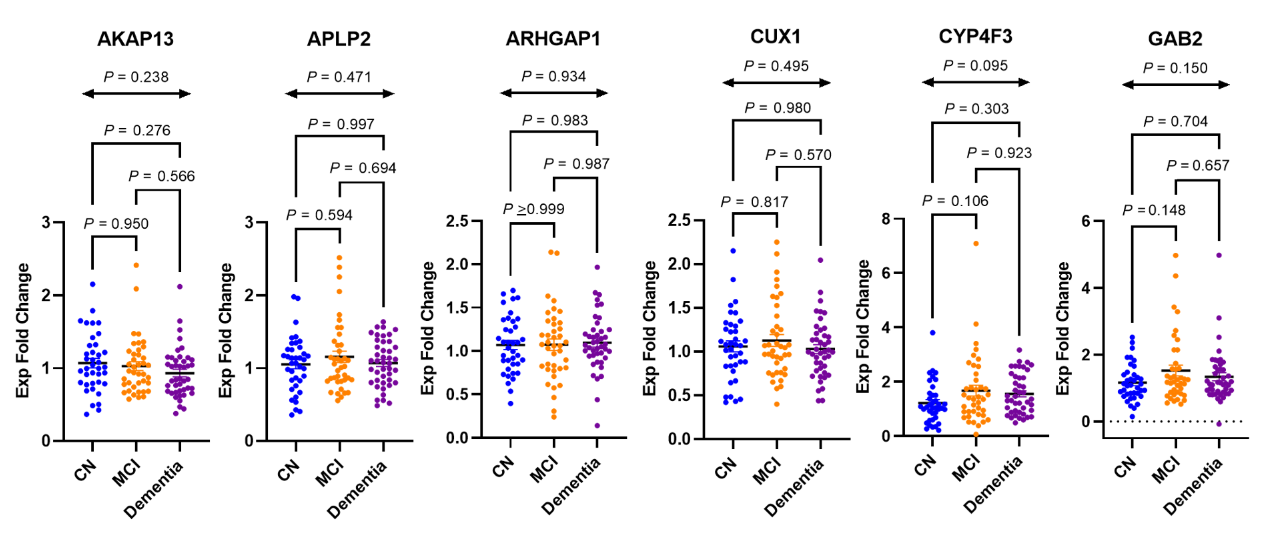
Figure S1.5. AD genes associated with memory database, not IL-7Rα^low^ aging** **genes**

**
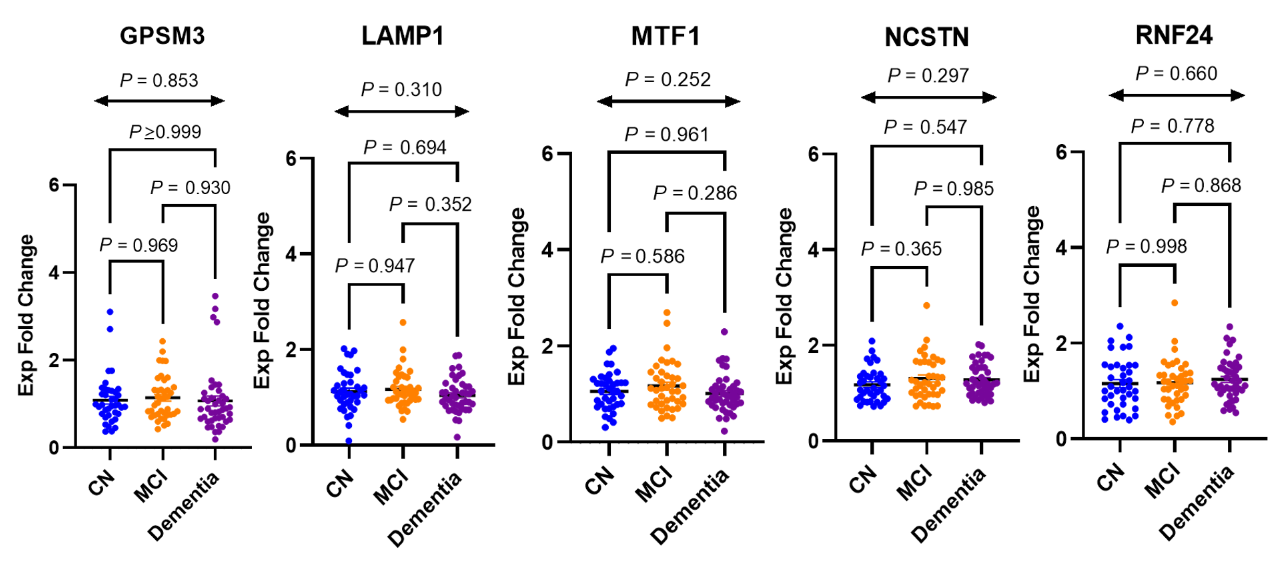
Figure S1.5. AD genes associated with memory database, not IL-7Rα^low^ aging** **genes (cont’d)**

**
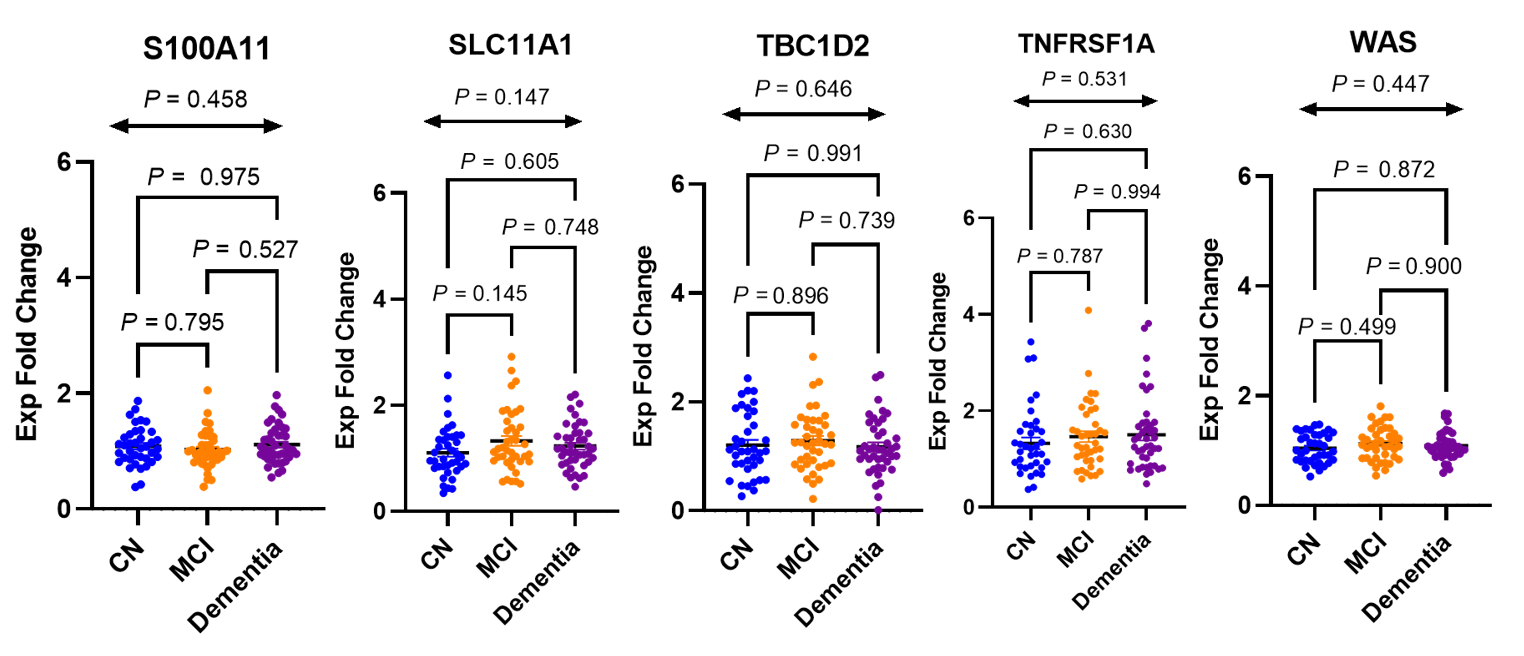
Figure S1.5. AD genes associated with memory database, not IL-7Rα^low^ aging** **genes (cont’d)**

**
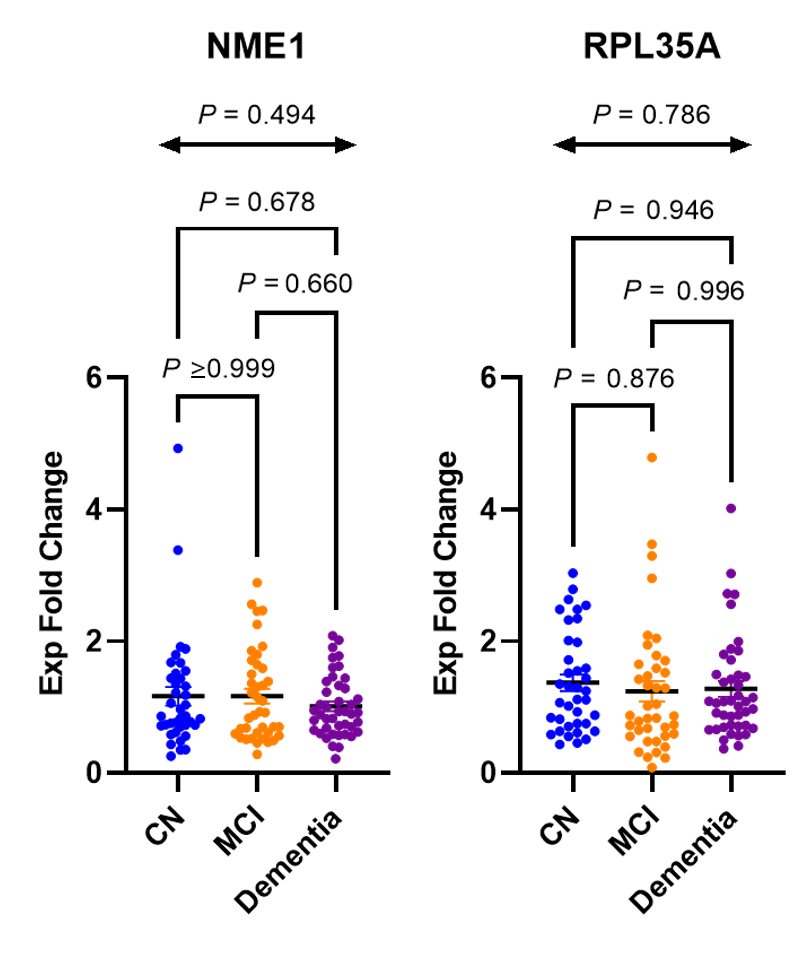
Figure S1.6. Genes and downregulated in 3 AD datasets; not IL-7Rα^low^ aging** **genes**

**
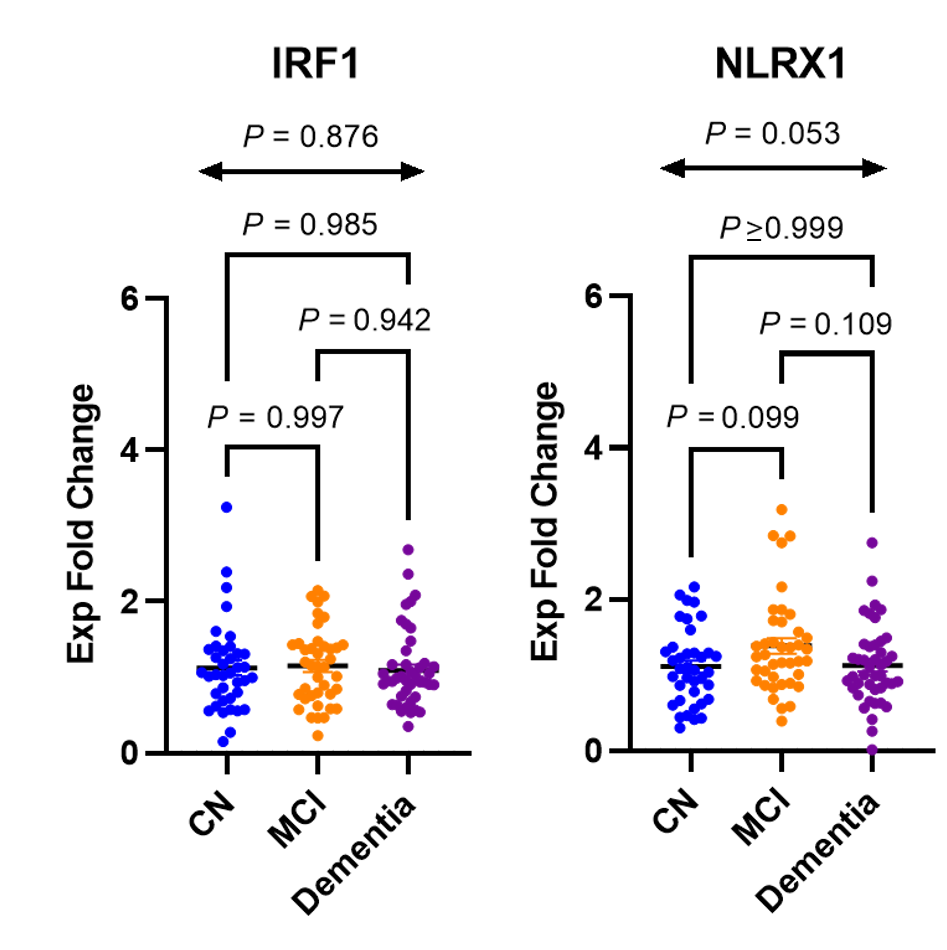
Figure S1.7. Inflammatory control genes**

Figures S1.1.-S1.7. Reverse transcription qPCR (RT-qPCR) analysis showing differentially expressed genes in peripheral blood of cognitively normal (CN) and AD patients with mild cognitive impairment (MCI) or dementia. Plots were shown with one-way ANOVA *P*-values (bidirectional arrow) and post-hoc multiple comparison testing adjusted *P-*values for genes that were not found to be differentially expressed between the three clinical groups. Abbreviations: AD, Alzheimer’s disease


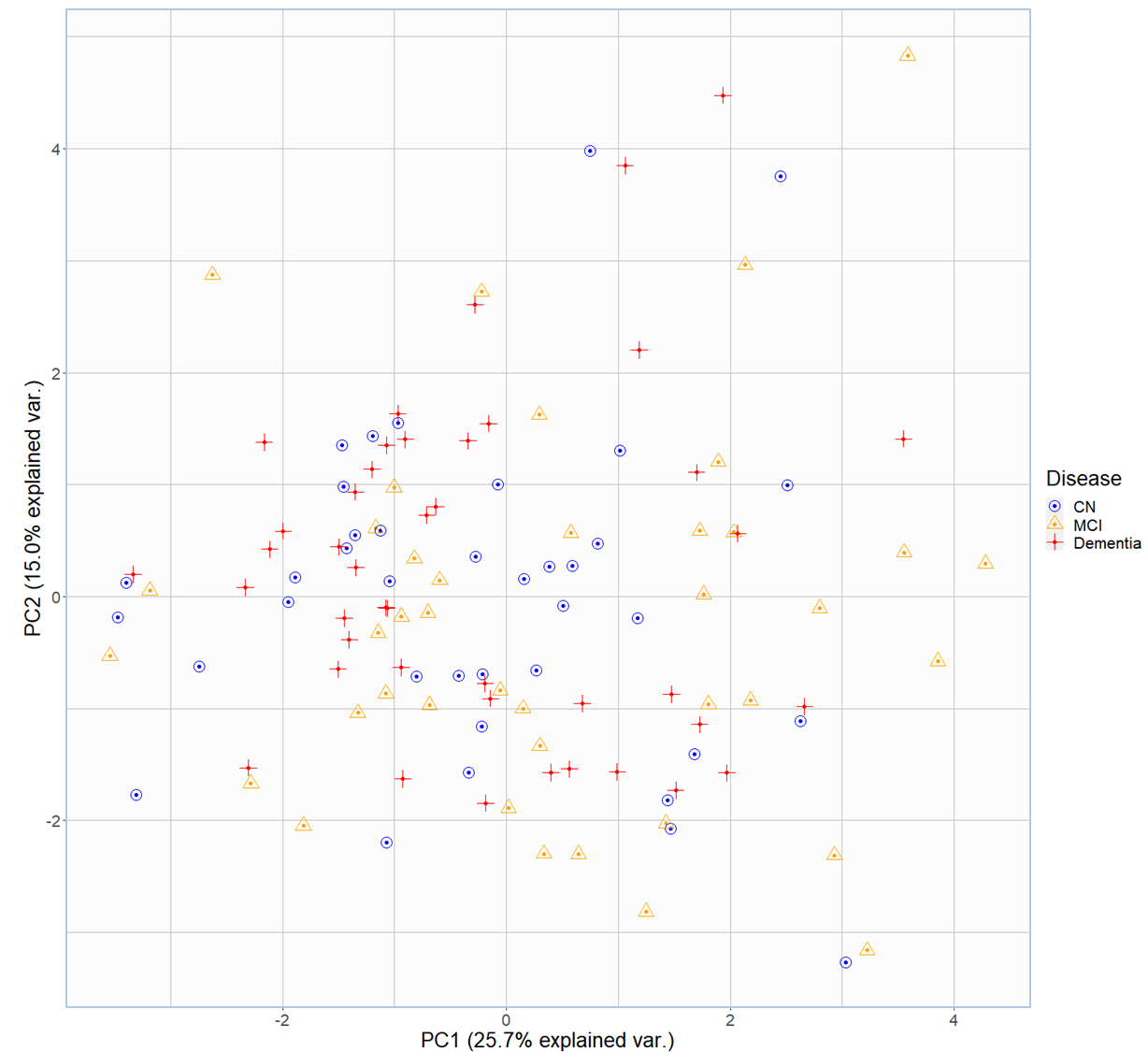
 **Figure S2. All Genes PCA Plot**

PCA plot generated using all gene target Z-scores calculated for all participants. Points on plot represent individual participants identified by clinical group. Blue circles represent CN, yellow triangles represent MCI, and red crosses represent dementia group participants. Abbreviations: PCA, principal component analysis; var, variance; CN, cognitively normal; MCI, mild cognitive impairment


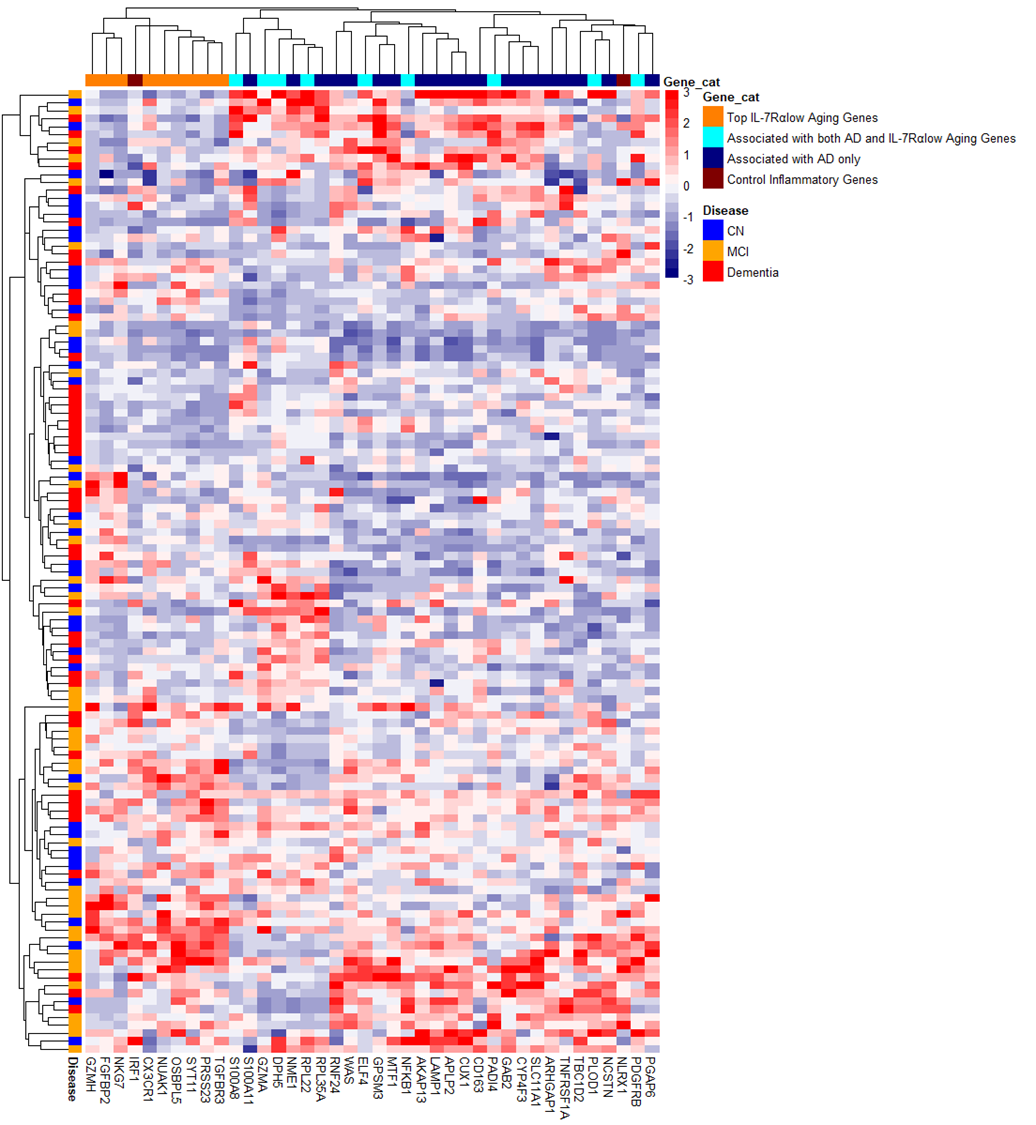


**Figure S3. Hierarchal Clustering Heatmap**

Unbiased hierarchal clustering of all gene categories according to differential gene expression of individual participants. Clustering is labeled according to gene category on the horizontal axis and clinical group on the vertical axis. Clustering is based on the “Euclidean” distance of gene target expression levels from one another. The blue arrow indicates a cluster with high expression levels of the top aging genes associated with IL-7Rα^low^ EM CD8^+^ T cells. Abbreviations: CN, cognitively normal; MCI, mild cognitive impairment

**
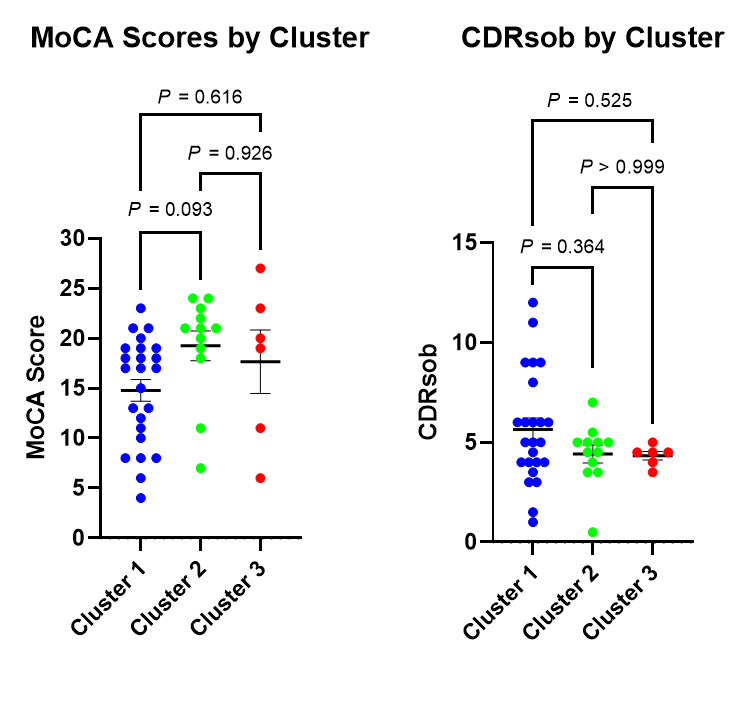
**

**Figure S4. MoCA and CDRsob Scores per Dementia Cluster**

MoCA and CDRsob scores plotted according to cluster designation in the dementia group. Abbreviations: MoCA, Montreal Cognitive Assessment; CDRsob, Clinical Dementia Rating scale sum of boxes

**Figure S5.1. Processing Speed Z-scores vs. Gene Expression Z-scores**


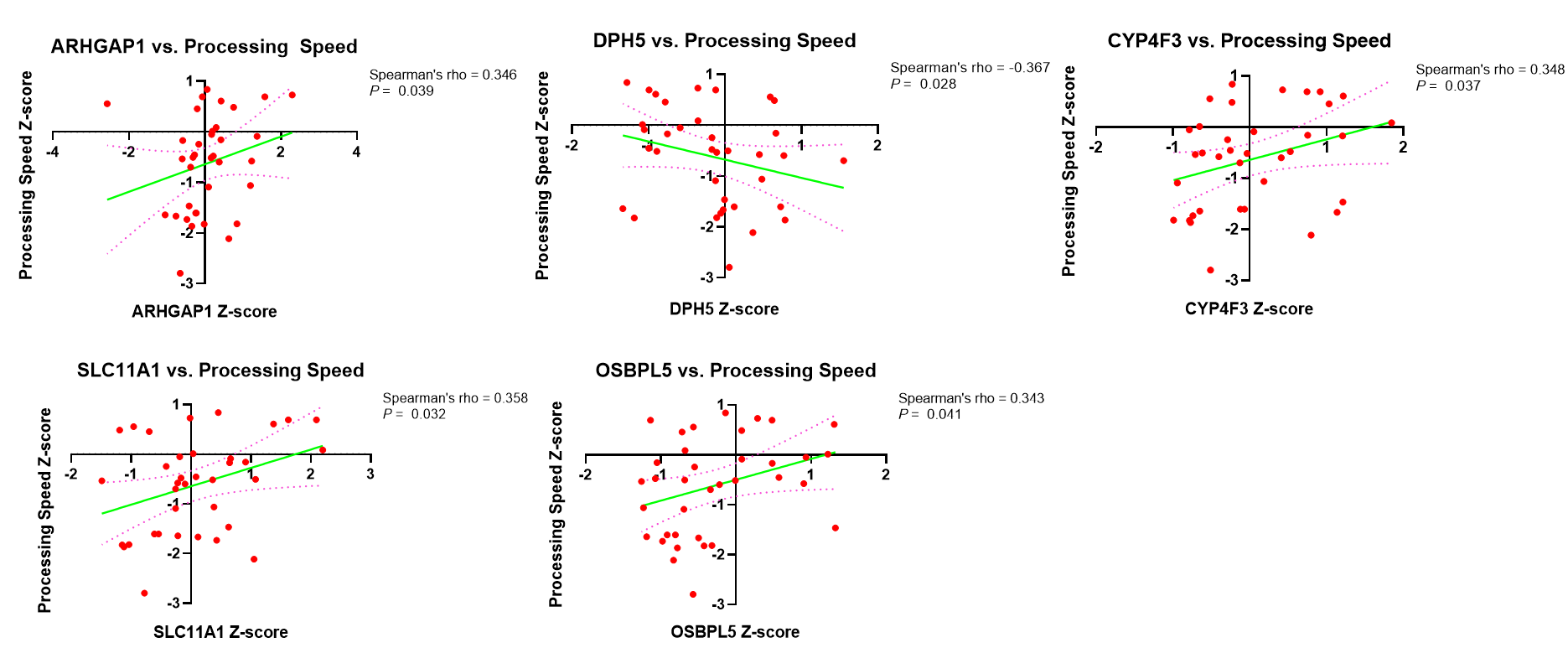

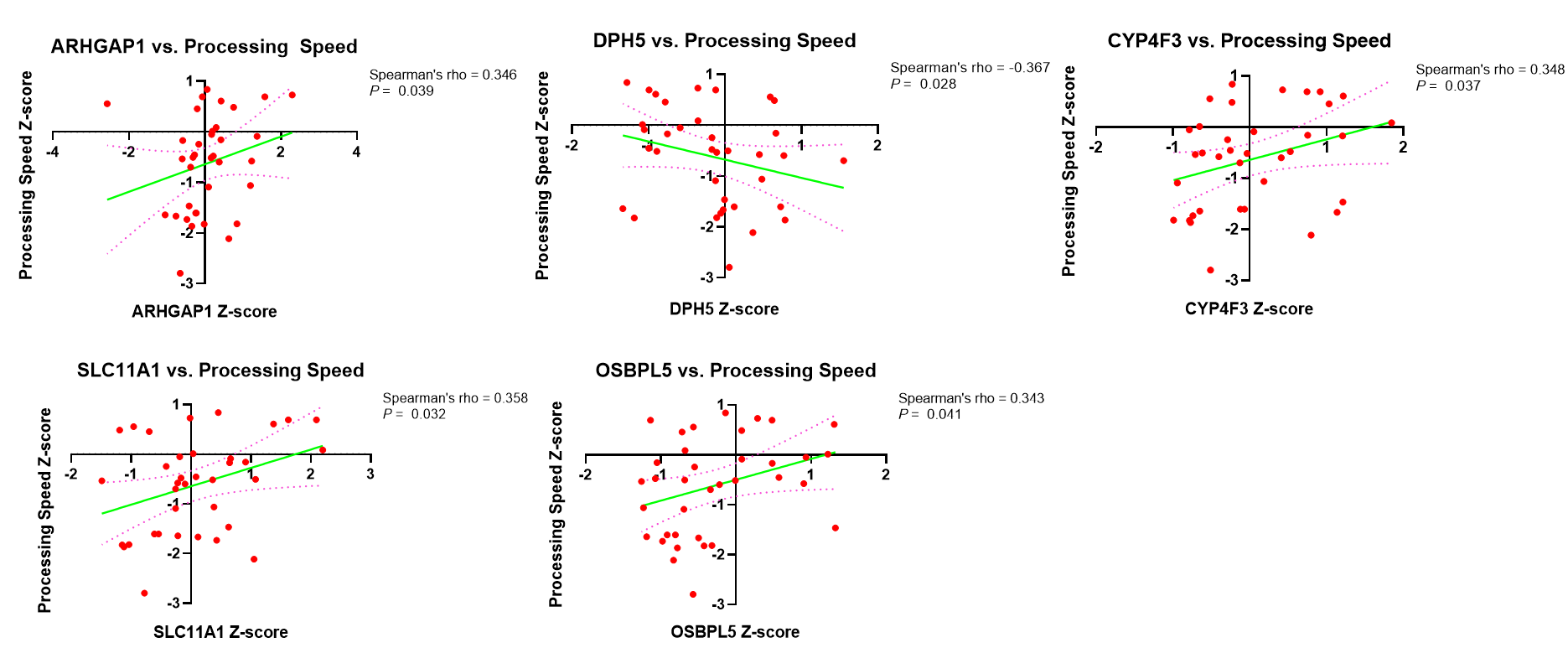


**Figure S5.2. Verbal Memory Z-scores vs. Gene Expression Z-scores**


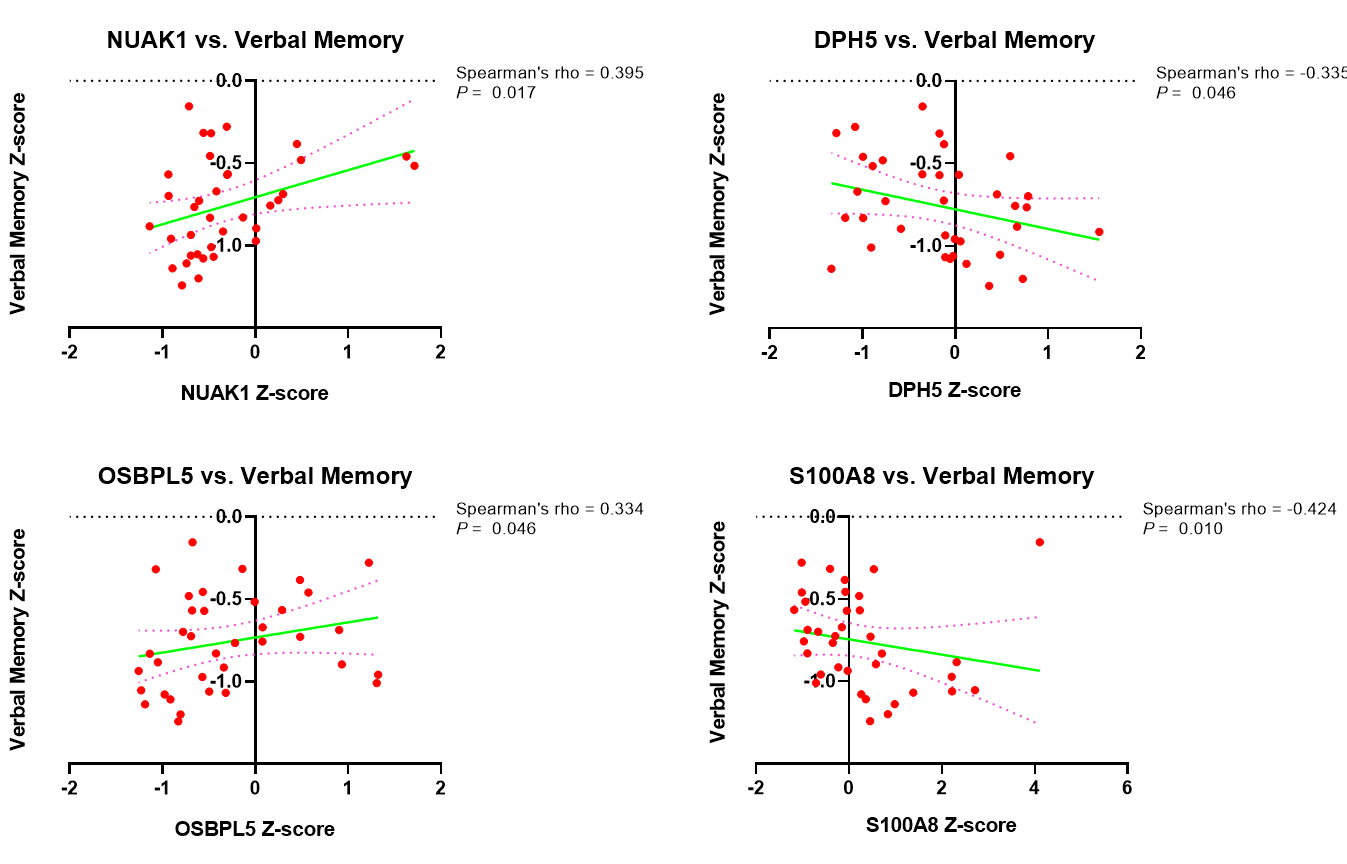


**Figure S5.3. Episodic Memory Z-scores vs. Gene Expression Z-scores**


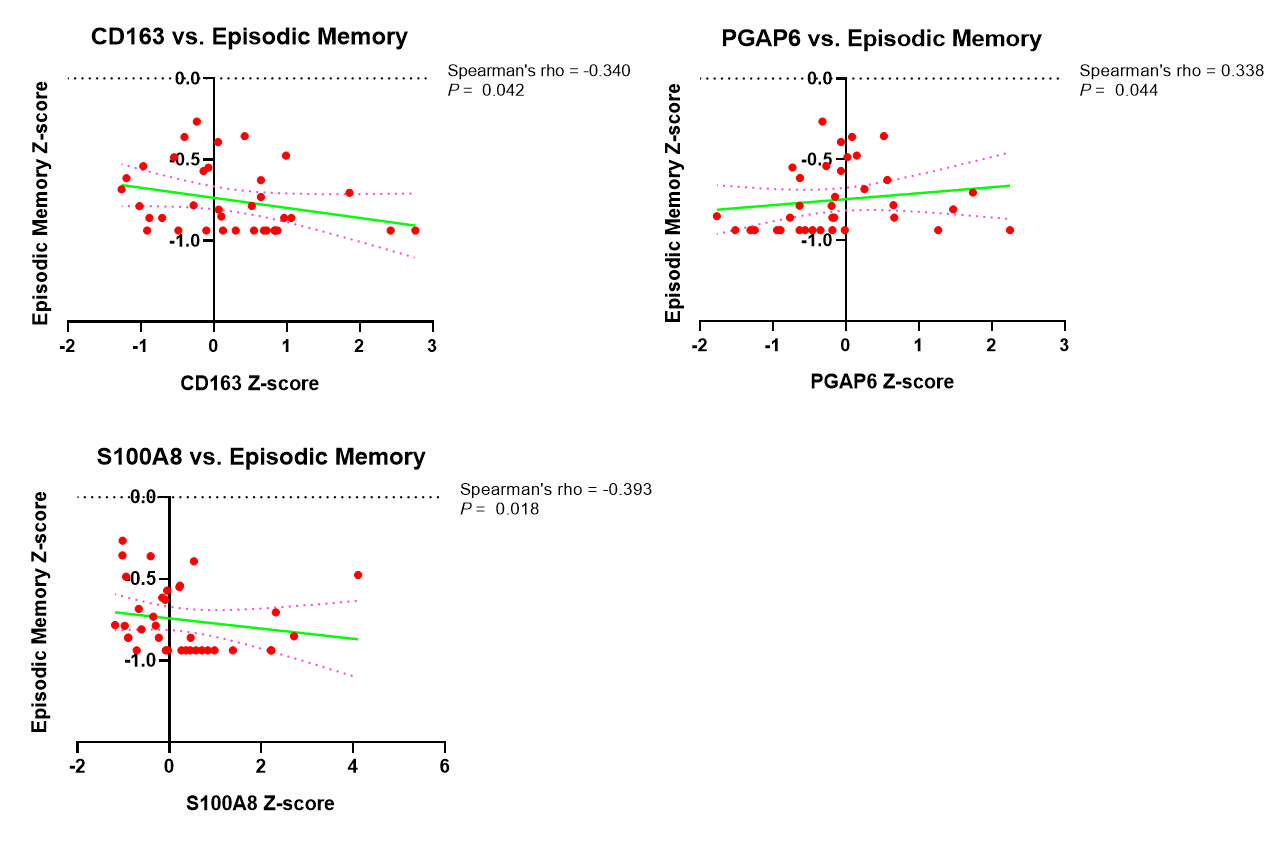


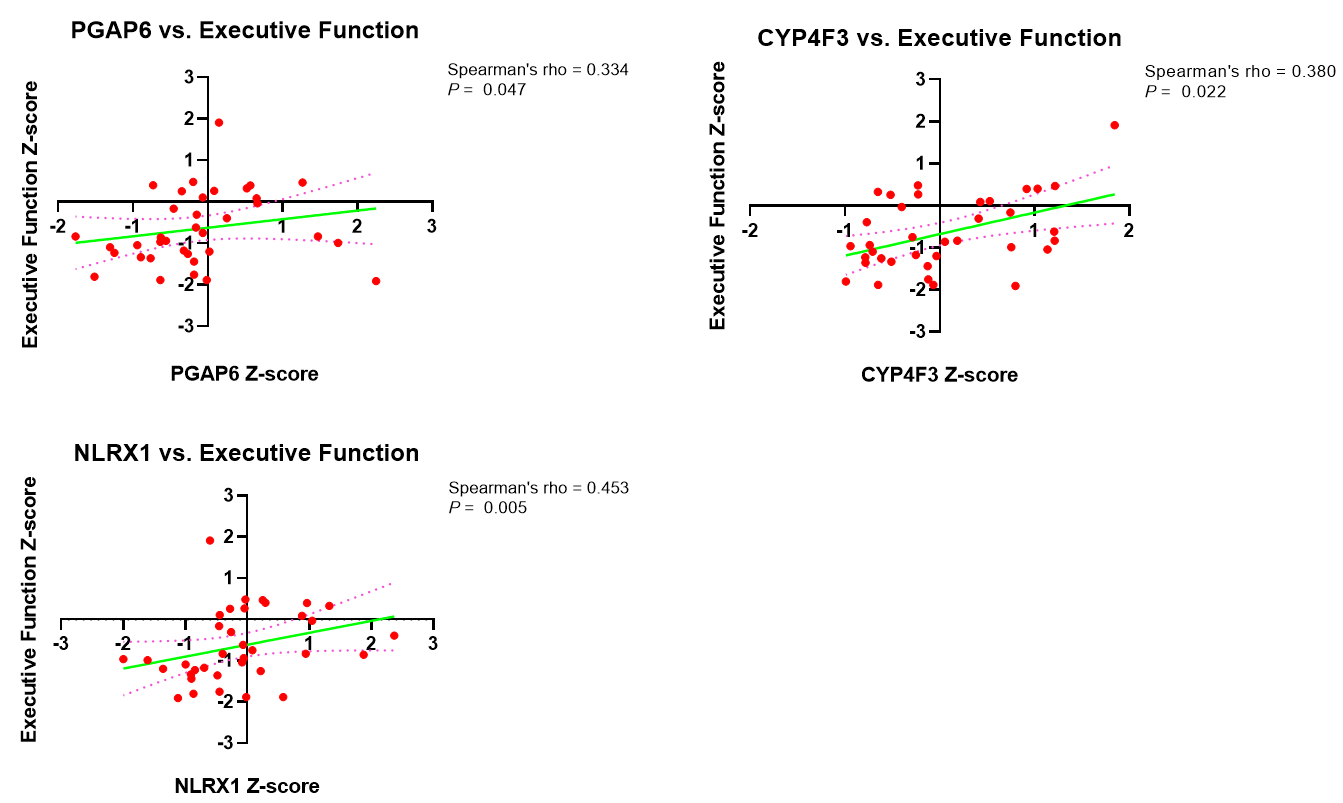
**Figure S5.4. Executive Function Z-scores vs. Gene Expression Z-scores**

**Figure S5.5. Language Z-scores vs. Gene Expression Z-scores**

**
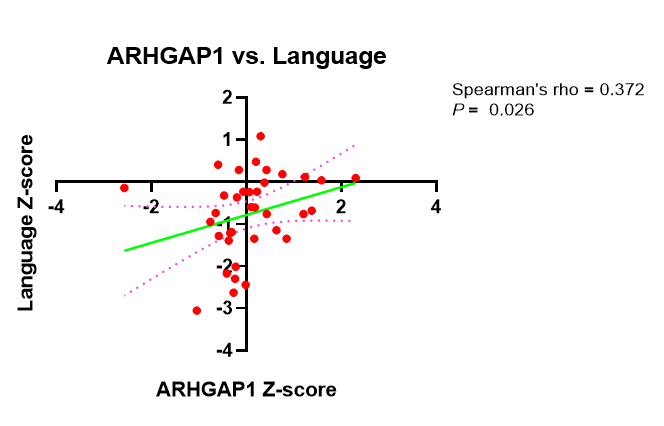
**

**Figure S5.6. Visuospatial Ability Z-scores vs. Gene Expression Z-scores**

Figures S5.1.-S5.6. Neuropsychological testing Z-scores of dementia participants for each cognitive domain composite plotted against gene expression Z-scores with statistically significant Spearman’s rho coefficients. Linear regression plots (green lines) with ± 2 SD (pink dots) were also visualized.

**Supplementary References**

1 Nachun, D. *et al.* Systems-level analysis of peripheral blood gene expression in dementia patients reveals an innate immune response shared across multiple disorders. *bioRxiv*, 2019.2012.2013.875112, doi:10.1101/2019.12.13.875112 (2019).

2 Sood, S. *et al.* A novel multi-tissue RNA diagnostic of healthy ageing relates to cognitive health status. *Genome Biol* **16**, 185, doi:10.1186/s13059-015-0750-x (2015).

3 Liberzon, A. *et al.* Molecular signatures database (MSigDB) 3.0. *Bioinformatics* **27**, 1739-1740, doi:10.1093/bioinformatics/btr260 (2011).

4 Ray, S. *et al.* Classification and prediction of clinical Alzheimer's diagnosis based on plasma signaling proteins. *Nature medicine* **13**, 1359-1362, doi:10.1038/nm1653 (2007).

5 Blalock, E. M. *et al.* Incipient Alzheimer's disease: microarray correlation analyses reveal major transcriptional and tumor suppressor responses. *Proc Natl Acad Sci U S A* **101**, 2173-2178, doi:10.1073/pnas.0308512100 (2004).

6 Wu, Z. *et al.* Role of the MEOX2 homeobox gene in neurovascular dysfunction in Alzheimer disease. *Nature medicine* **11**, 959-965, doi:10.1038/nm1287 (2005).

7 Niculescu, A. B. *et al.* Blood biomarkers for memory: toward early detection of risk for Alzheimer disease, pharmacogenomics, and repurposed drugs. *Mol Psychiatry* **25**, 1651-1672, doi:10.1038/s41380-019-0602-2 (2020).

8 Park, H. J. *et al.* Transcriptomic analysis of human IL-7 receptor alpha (low) and (high) effector memory CD8(+) T cells reveals an age-associated signature linked to influenza vaccine response in older adults. *Aging Cell* **18**, e12960, doi:10.1111/acel.12960 (2019).

9 Schmittgen, T. D. & Livak, K. J. Analyzing real-time PCR data by the comparative C(T) method. *Nat Protoc* **3**, 1101-1108, doi:10.1038/nprot.2008.73 (2008).

10 Gentleman, R. C. *et al.* Bioconductor: open software development for computational biology and bioinformatics. *Genome Biol* **5**, R80, doi:10.1186/gb-2004-5-10-r80 (2004).

11 Stacklies, W., Redestig, H., Scholz, M., Walther, D. & Selbig, J. pcaMethods—a bioconductor package providing PCA methods for incomplete data. *Bioinformatics* **23**, 1164-1167, doi:10.1093/bioinformatics/btm069 (2007).

12 Leek, J. T. *et al.* sva: Surrogate Variable Analysis. *R package version 3.42.0.* (2021).

13 Johnson, W. E., Li, C. & Rabinovic, A. Adjusting batch effects in microarray expression data using empirical Bayes methods. *Biostatistics* **8**, 118-127, doi:10.1093/biostatistics/kxj037 (2006).

14 Reimand, J. *et al.* Pathway enrichment analysis and visualization of omics data using g:Profiler, GSEA, Cytoscape and EnrichmentMap. *Nature Protocols* **14**, 482-517, doi:10.1038/s41596-018-0103-9 (2019).

15 Shannon, P. *et al.* Cytoscape: a software environment for integrated models of biomolecular interaction networks. *Genome Res* **13**, 2498-2504, doi:10.1101/gr.1239303 (2003).

16 Merico, D., Isserlin, R., Stueker, O., Emili, A. & Bader, G. D. Enrichment map: a network-based method for gene-set enrichment visualization and interpretation. *PLoS One* **5**, e13984, doi:10.1371/journal.pone.0013984 (2010).

17 Oesper, L., Merico, D., Isserlin, R. & Bader, G. D. WordCloud: a Cytoscape plugin to create a visual semantic summary of networks. *Source Code Biol Med* **6**, 7, doi:10.1186/1751-0473-6-7 (2011).

18 Kucera, M., Isserlin, R., Arkhangorodsky, A. & Bader, G. D. AutoAnnotate: A Cytoscape app for summarizing networks with semantic annotations. *F1000Res* **5**, 1717, doi:10.12688/f1000research.9090.1 (2016).

19 Manimaran, S. *et al.* BatchQC: interactive software for evaluating sample and batch effects in genomic data. *Bioinformatics* **32**, 3836-3838, doi:10.1093/bioinformatics/btw538 (2016).

20 Ritchie, M. E. *et al.* limma powers differential expression analyses for RNA-sequencing and microarray studies. *Nucleic Acids Res* **43**, e47, doi:10.1093/nar/gkv007 (2015).

21 R package 'corrplot': Visualization of a Correlation Matrix v. Version 0.92 (2021).

22 Le, S., Josse, J. & Husson, F. FactoMineR: An R Package for Multivariate Analysis. *Journal of Statistical Software* **25**, 1-18, doi:10.18637/jss.v025.i01 (2008).

23 ggbiplot: A ggplot2 based biplot v. R package version 0.55 (2011).

24 pheatmap: Pretty Heatmaps v. R package version 1.0.12 (2019).

25 Wickham, H. *et al.* Welcome to the Tidyverse. *Journal of Open Source Software* **4**, 1686, doi:10.21105/joss.01686 (2019).

26 cluster: Cluster Analysis Basics and Extensions v. R package version 2.1.2 (2021).
